# Plasma Control: A Review of Developments and Applications of Plasma Medicine Control Mechanisms

**DOI:** 10.3390/plasma7020022

**Published:** 2024-05-27

**Authors:** Jonathan E. Thomas, Katharina Stapelmann

**Affiliations:** Department of Nuclear Engineering, North Carolina State University, Raleigh, NC 27695, USA

**Keywords:** plasma control, predictive control, cold atmospheric plasma, machine learning, plasma processing, dielectric barrier discharge, atmospheric pressure plasma jet, neural network, plasma medicine, plasma oncology

## Abstract

Cold atmospheric plasmas (CAPs) within recent years have shown great promise in the field of plasma medicine, encompassing a variety of treatments from wound healing to the treatment of cancerous tumors. For each subsequent treatment, a different application of CAPs has been postulated and attempted to best treat the target for the most effective results. These treatments have varied through the implementation of control parameters such as applied settings, electrode geometries, gas flow, and the duration of the treatment. However, with such an extensive number of variables to consider, scientists and engineers have sought a means to accurately control CAPs for the best-desired effects in medical applications. This paper seeks to investigate and characterize the historical precedent for the use of plasma control mechanisms within the field of plasma medicine. Current control strategies, plasma parameters, and control schemes will be extrapolated through recent developments and successes to gain better insight into the future of the field and the challenges that are still present in the overall implementation of such devices. Proposed approaches, such as data-driven machine learning, and the use of closed-loop feedback controls, will be showcased as the next steps toward application.

## 1. Introduction

Since the discovery of plasmas, the possibility for potential applications have been numerous. For most of these applications, control of the plasma is key to a successful introduction and transition to industry. In order to control plasma, variables need to be defined and measured in a practical way to have an impact on the process. Then, different control schemes can be employed ranging from a simple endpoint detection to an active process control. With the emergence of different control concepts in combination with Artificial Intelligence (AI)/Machine Learning (ML), the opportunities for controlling plasmas for new applications like plasma medicine have become feasible. In fact, several plasma medicine applications have started the transition to clinics [1]. By utilizing established control concepts and contemporary means of predictive control, plasmas are set to play a crucial role in medical applications. Yet, a focused approach in terms of control schemes and processes is still needed for specific plasma therapies and treatments moving forward.

Control systems are not a new concept to the medical field as several closed-loop control devices have actively been demonstrated in practice. One such device is an epilepsy control monitor that uses implantable multi-electrode arrays and amplifiers to record electrical signals used to predict and even prevent a seizure [2]. Another medical control system proposed, uses an insulin adaptive predictive controller for artificial pancreas systems. The control system works to estimate the current insulin concentration in a patient, and computes the optimal dosage of insulin delivery to the patent without prior user-provided information on carbohydrate consumption [3]. These same methodologies practiced in medical devices today can actively be applied to medical plasma control research as well.

Major obstacles are still present in defining a centralized control system for plasma medicine applications. The complexity of defining a plasma “dose” or tailored treatment method lies in the dynamic nature of plasma substrate interactions. Plasma interactions, during surface treatments of biological tissue and liquid interactions, is a complex control problem that must be evaluated from multiple perspectives. Three overarching factors of generation, transport, and translation of plasma species occur simultaneously across plasma surface interactions, and are crucial areas that must be investigated for controlling any plasma medicine applications going forward. For example, generation of reactive species by plasma is tied to the electrical circuit that is created between the plasma and the substrate involved. Power delivered to the substrate depends on the overall circuit impedance, which, in turn, is affected by the electrical properties of the target being treated [4]. The transport of generated reactive species can also be impacted, retroactively, due to plasma chemistry changes in the gas phase by more or less deposited power [5]. Changing chemical interactions by the transported species with the substrate can alter the dielectric properties of the substrate [6,7] impacting surface charge and sheath formation [8]. Lastly, the duration of time to which each of these interactions occurs with the substrate can significantly affect the plasma and the substrate treated over time [9].

In this review, we aim to introduce an overview on control concepts, and how they have been successfully implemented in plasma applications, despite the aforementioned challenges involved in controlling plasmas when interacting with substrates. Since many researchers are new to the field of control engineering, a brief history and evolution of the field will be provided before we dive into the literature of plasma control concepts. To provide further context into how plasma can be controlled, we will also discuss what we can learn from established control practices in etching and deposition and fusion for future utilization in the field of plasma medicine. Examples of plasma devices being used for medical applications in clinics today was demonstrated by Brehmer et al. where evidence of safe and effective CAP treatment in patients with chronic venous leg ulcers using the PlasmaDerm^®^ VU-2010, Cinogy, Duderstadt, Germany plasma device was shown [10]. Isbary et al. showcased that treatment with the MicroPlaster^®^, (ADTEC Plasma Technology Co. Ltd., Hiroshima, Japan and London, UK) plasma device decreased bacterial load for chronic wounds in patients and promoted wound healing [11]. With plasma medical devices now serving as a viable treatment method, the need for specified protocols and precision control are essential moving forward. Providing a background understanding into these current control practices, will give greater context into the recently developed strategies like real-time control and machine learning algorithms. We thus will endeavor to present the current challenges associated with the control of medical plasma devices, and provide future recommendations to advance the field.

## 2. An Overview on Control Concepts

### 2.1. A Brief History of Control Engineering

Control engineering and control systems are in play in the modern era in most aspects of daily life, with many of these control systems scarcely being recognized by the general public. The earliest known example of a control system device was a water clock created by the Greek physicist and inventor, Ctesibius of Alexandria, Egypt, around 270 B.C. [12]. The water clock kept time by dripping water into a container at a constant rate, to regulate a float connected to a pointer, to mark the passage of hours [12]. This type of control device is known today as the first feedback control mechanism on record.

**Figure 1.**
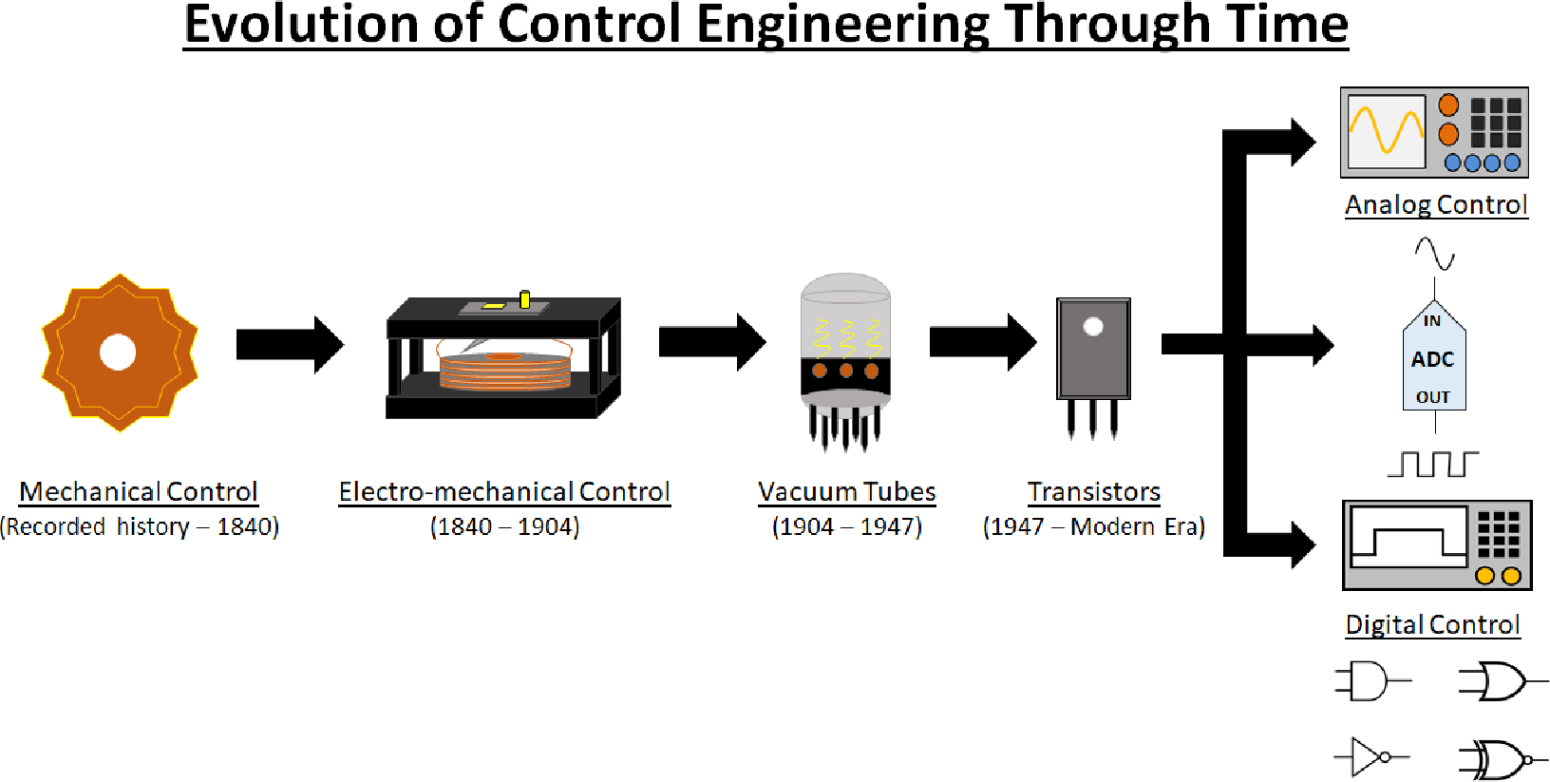
The transition of control engineering through time defined by the devices that made each era of control possible. Early records of mechanical control were depicted as early as ancient Egypt, with electro-mechanical control taking its place with the onset of linking electricity and magnetism. Vacuum tubes were followed which allowed for amplification of circuit signals and faster response times. Consolidation of device foot-prints was then achieved by the innovation of the transistor which lead to two branching methods of control used today being analog and digital control.

On a broad scale, control systems are any system that uses inputs, and utilizes this “information” to produce a regulated desired output. An evolution of the control systems through time is shown schematically in Figure 1. As was demonstrated by the water clock, most early applications of control were purely mechanical in nature, until Hans Christian Ørsted in 1820 discovered that the flow of electric current creates a proportional magnetic field, confirming the connection between electricity and magnetism [13]. This connection would give rise, amongst other things, to the second industrial revolution where electricity became a force of technological innovation, with the idea of transporting and generating electricity being solved by the 1890s [14]. The idea of controlling this electricity for practical uses would arise in the form of Samuel E. B. Morse’s telegraph in 1840 [15]. The telegraph became the first major use of a electromagnetic device that would branch into what is known today as electro-mechanical control. Electro-mechanical control saw the development of the first timers, controllers, on/off switches, and relays. Such devices, with low power consumption, could provide a means to controlling more complicated systems such as power plants, automobiles, and household appliances like a refrigerator. These systems would mainly operate under the principle that electricity would be provided to manipulate a mechanical operation through either isolating or sending electricity to the desired part of the circuit for a specific result.

Electro-mechanical operation would be the dominate form of control until the onset of the vacuum tube. By eliminating mechanical inertia, vacuum tubes were able to operate millions of times faster than electro-mechanical devices and made it possible to generate, amplify or control electrical signals in larger ranges [16]. Vacuum tubes used electrostatic, and in some cases, magnetic forces to directly control the flow of electrons from a negatively charged (and heated) cathode to a positively charged plate [16]. The invention of the vacuum tube by John Ambrose Fleming would provide a means for signal amplification and current rectification that was yet unattainable at the time [17]. The new method of manipulating electron flow was instrumental into the development of sound and video recording, television, and radio systems. Efforts to develop a solid state device that directly controls the flow of electrons without the inconvenience of heating a cathode or maintaining a vacuum envelope began in the 1930s, and would lead to the first solid-state device known as a transistor in the late 1940s [16]. Solid-state would begin to define all integrated circuits without moving parts that could manipulate and control the flow of electrons following the invention of the transistor. John Bardeen and Walter Brattain discovered the transistor, in an effort to have a similar device to the vacuum tube that could operate at higher frequencies and allow for more control of a given circuit [18]. Acting as a switch and amplifier, this solid-state alternative would eliminate the need for a vacuum and not overheat at a reduced footprint. The electrical current would flow through silicon crystals that were developed as either n- or p-type silicon. This designation referred to either a positive (p-type) or negative (n-type) rectification of the signal [18]. Once the benefits of the transistor became evident, devices that once used to contain vacuum tubes soon began being replaced by solid-state controlled versions. The transistor radio, computers, and mobile phones all became realities due to the reduced size, energy efficiency, and means of control the transistor allowed. The modern era sees transistors as the basis for control engineering in two domains today: analog and digital control.

The main difference in the two control methods is in how the signals are transmitted. Analog control, today, handles continuous voltage and current signals at different amplitudes and frequencies. This type of control is best utilized for continuous operational control in devices that primarily utilize filters and amplifiers. Pressure regulation, temperature, audio, and motor control are major areas where continuous monitoring of a signal through analog control is still in use today. Though not as discretely accurate, analog control is more cost-effective, and has quicker response times that are not present when a control method utilizes an analog-to-digital converter for instance. Digital control on the other hand was the next step in evolution from analog control in which digital control is an on-off signal that is transmitted as either a “0” for off and a “1” for on. This definition will be the most familiar to most readers as it is the backbone for most control systems today. Digital control is superior in its precision, flexibility, and its advanced control capabilities. In fact, most control systems, if utilizing digital control, will likely employ an analog-to-digital converter within their control systems to have increased means of control over the system. For example, energy metering will employ an analog method to capture the voltage and current utilization, but will process the digital measurements for recording the numerical amount of power used by a household for billing or load profiling. Digital control also has the advantage in terms of utilizing a form of mathematics known as control logic or Boolean algebra. Boolean algebra is the foundation of digital circuit design and allows for the combination of different logic elements (i.e, AND, OR, NAND, NOR, XOR, XNOR, NOT, etc.) to allow for complex control schemes otherwise not available to analog circuitry. Control schemes will usually include block diagrams that consist of many of these gates in each element to create an overall view of the control application at hand.

Solid-state electronics ushered the microelectronic and control domain we see today. Ironically enough, most of the control capability that is available today is thanks to the manufacturing of more advanced transistors brought about by the use of plasma based manufacturing. Plasma etching and deposition has allowed for the creation of integrated circuits down to a size of 3 nm with 2 nm sized chips expected to arrive in 2025 [19]. By understanding where the control industry has come from with the relationship of electricity and magnetism to create control systems, using the same principles to create nanometer-sized solid-state electronics, the future of plasma based control soon has the possibility of becoming a staple in the medical field as its next horizon.

### 2.2. Control Systems

#### 2.2.1. Open-Loop and Closed-Loop Systems

**Figure 2.**
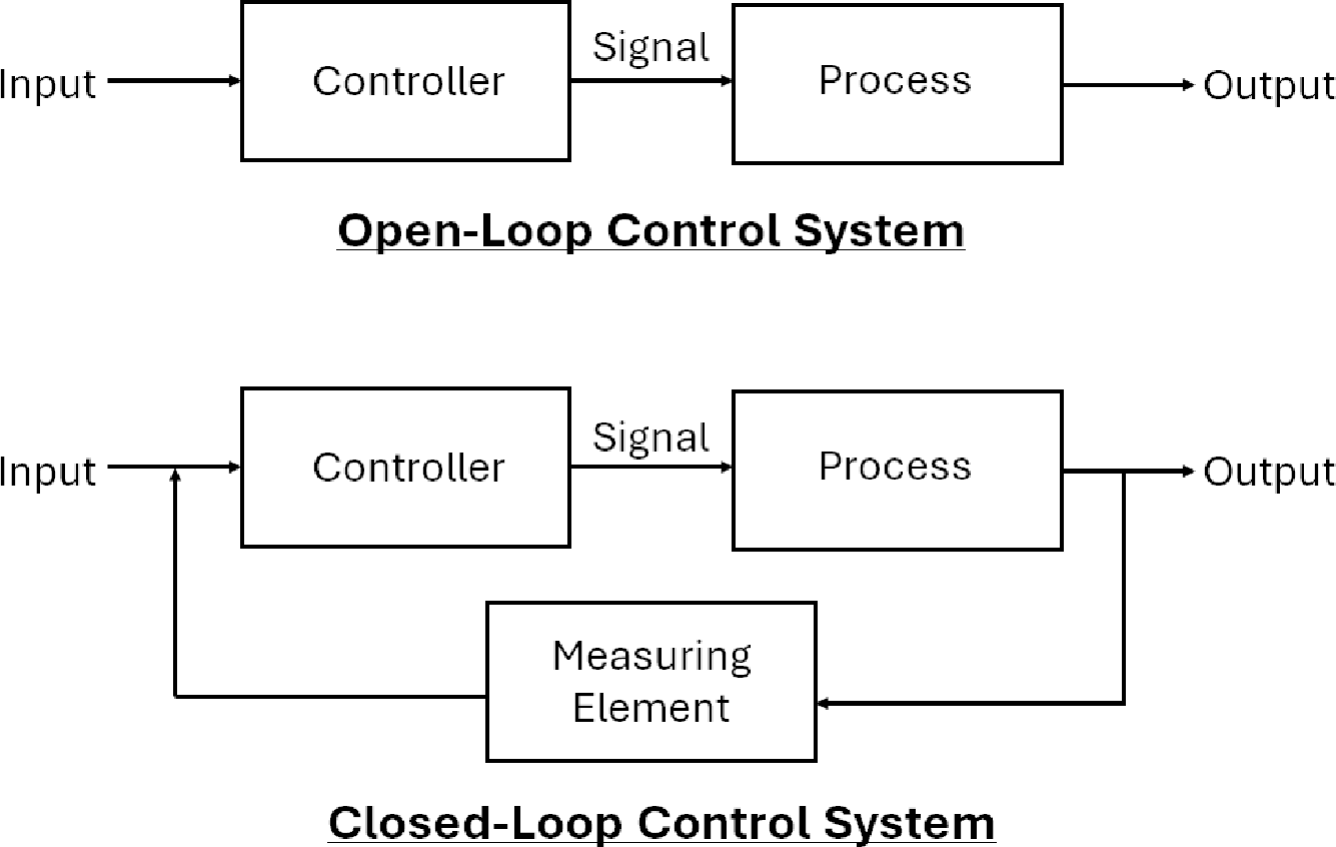
Overview block diagram depicting the differences in open-loop control systems versus closed-loop control systems.

After a brief history of control engineering, the aspects that comprise and are needed for modern plasma derived control will now be discussed. Control systems are generally divided into two main types: an open-loop control strategy, and closed-loop control strategy. An open-loop control strategy has a set desired output, but does not utilize the output to adjust or contribute to the overall output. On the other hand, in a closed-loop control strategy, the output depends on the process output as the produced output is used to control the input for the next loop. Open-loop control strategies are used in most household appliances like a washing machine or dishwasher, while closed-loop control strategies are used in air conditioning systems, for example. With both control system strategies, a controller is needed to manage the desired set-points and regulate that the desired output is met. A visual depiction of an open-loop and closed-loop control strategy can be seen in Figure 2.

**Figure 3.**
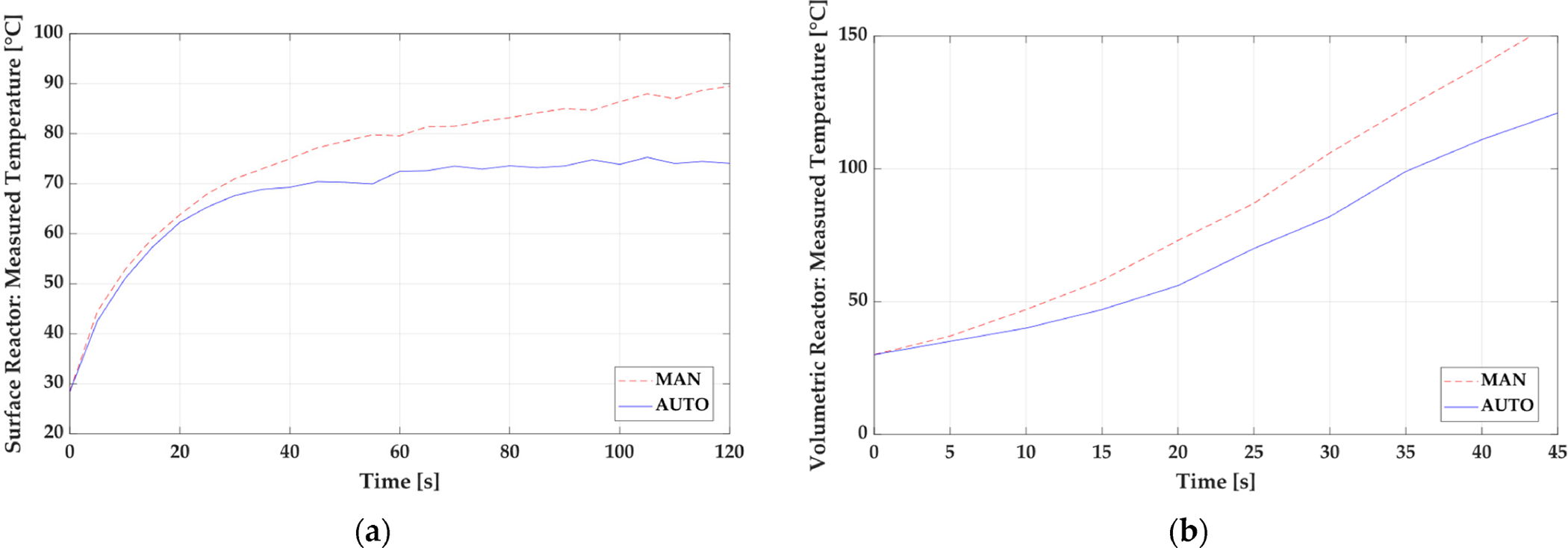
Measured reactor temperature of a surface DBD (**a**) and a volume DBD (**b**) comparing an open-loop control strategy (MAN) and a closed-loop control strategy (AUTO). Reprinted/adapted with permission from Ref. [20]. Copyright 2022, Authors. Licensee MDPI, Basel, Switzerland. Image reprinted/adapted from an open access article distributed under the terms and conditions of the Creative Commons Attribution (CC BY) license.

**Figure 4.**
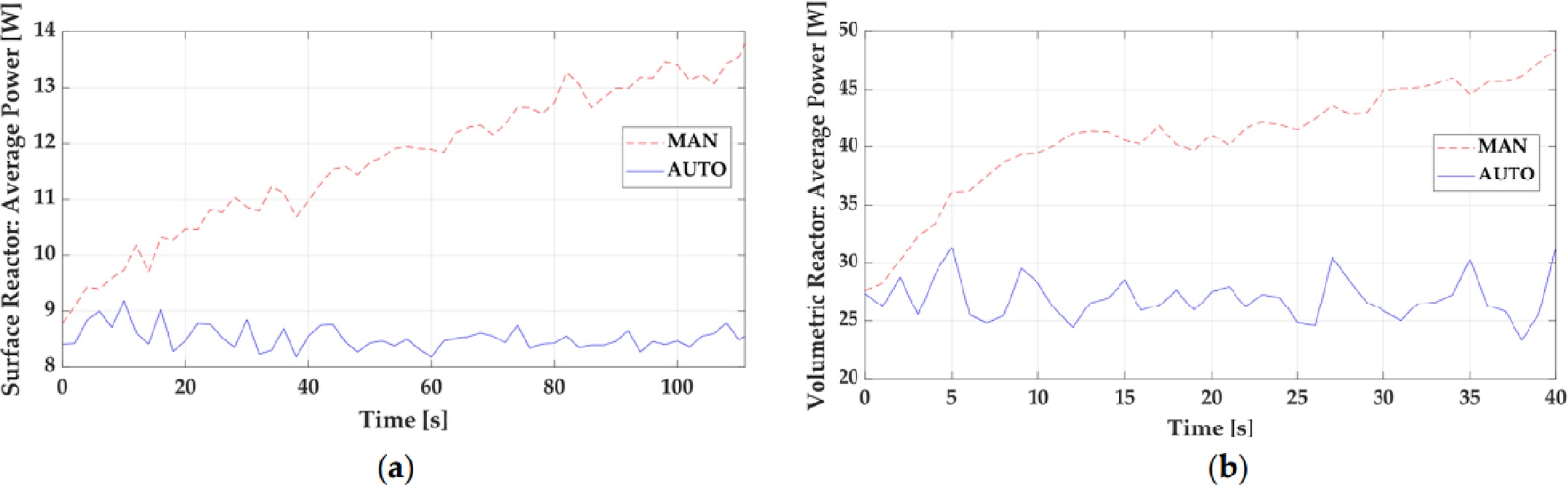
Measured average power of a surface DBD (**a**) and a volume DBD (**b**) comparing an open-loop control strategy (MAN) and a closed-loop control strategy (AUTO) Reprinted/adapted with permission from Ref. [20]. Copyright 2022, Authors. Licensee MDPI, Basel, Switzerland. Image reprinted/adapted from an open access article distributed under the terms and conditions of the Creative Commons Attribution (CC BY) license.

A comparison of the operational difference in both open-loop and closed-loop control strategies for plasma systems was highlighted by Neretti et al. in a surface and volume dielectric barrier discharge (DBD) experimental setup [20]. For both, plasma medicine and industrial plasma applications, there is a technical need for reliable and reproducible treatments, which require the discharge parameters to be actively controlled [20]. Therefore, Neretti et al. evaluated the temperature and average deposited plasma power to a substrate at 6 ms intervals where the applied voltage could be adjusted by the input DC voltage. The proposed control strategy to actively sample and perform diagnostic calculations in a real-time evaluation was performed by an Arduino DUE micro-controller [20]. As shown in Figures 3 and 4, for active and accurate power and temperature control of the plasma process, a closed-loop control strategy is superior to the open-loop control strategy to maintain the desired output parameters.

The performed measurements by Neretti et al. show that the implementation of the described control strategy allows the reactor temperature to be decreased by 20% (surface reactor) and by 30% (volumetric reactor) compared to when the power supply is operated in an open-loop mode [20]. Open and closed-loop control systems each have different variations in their design, but closed-loop control strategies are the basis for more advanced control for a consistently changing system like plasmas. In fact, closed-loop systems that are using different controllers will normally be dubbed simply as feedback controllers. Feedback controllers are usually operated in proportional (P) control, integral (I) control, or derivative (D) control, with a combination of the three being the basis for more advanced control schemes operating by using an error signal to reach a desired setpoint. Classical controllers consist of these most commonly used control techniques, such as on/off control and P, PI, and PID control [21].

#### 2.2.2. PID Controller

**Figure 5.**
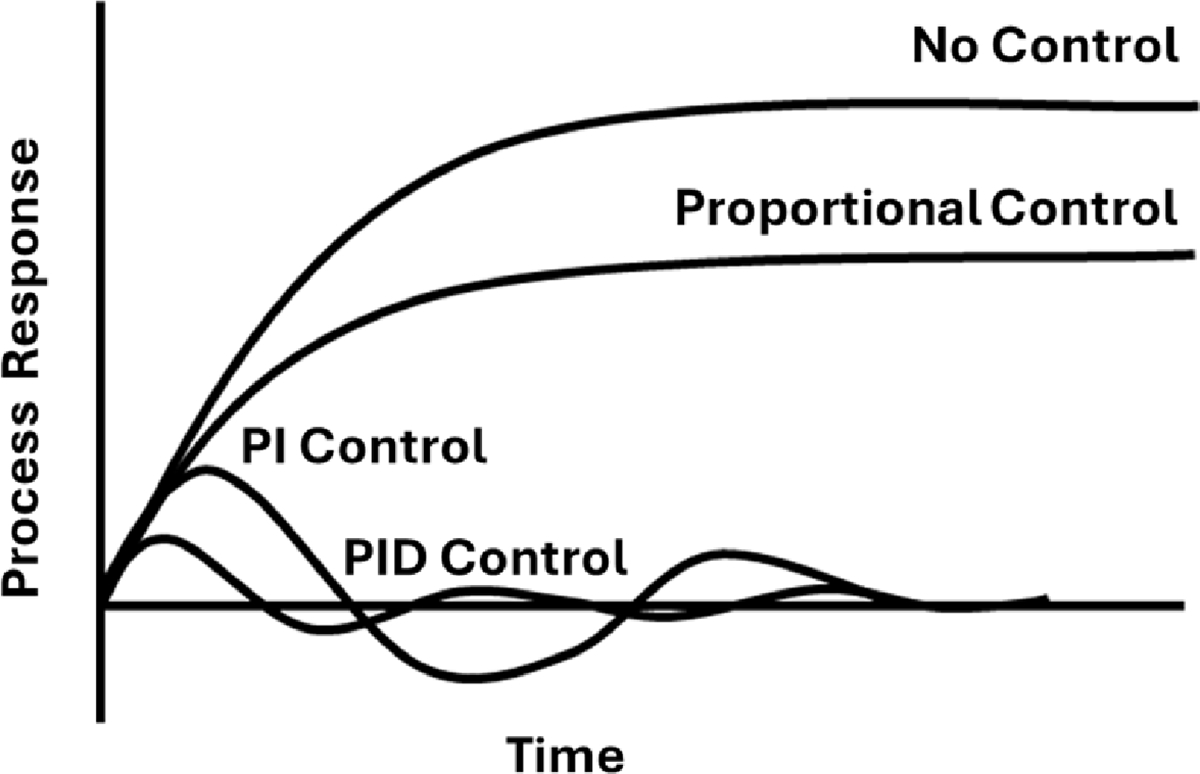
Typical process responses with feedback control for P, PI, and PID controller over time.

Figure 5 shows the typical responses over time for proportional control, PI, and PID controllers, and will be discussed in detail to provide context into how each of the controllers components improve the transient response of a system.

**Figure 6.**
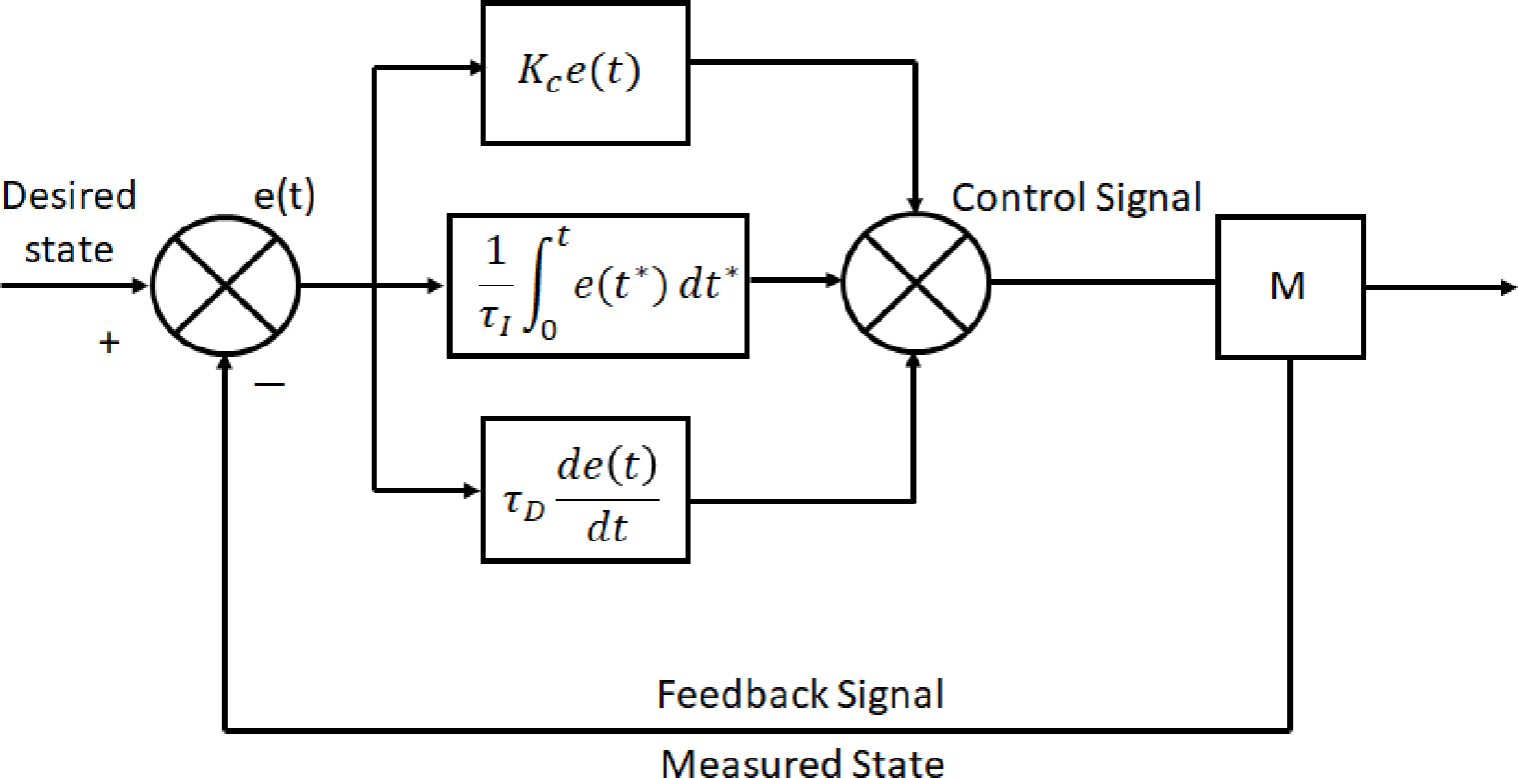
Block diagram showcasing the parallel form of PID controller.

A proportional controller’s main objective is to reduce the error of the systems signal to zero. As the mode suggests, the error signal is proportional to the controller output. The error signal is given by Equation (1),

(1) $$e(t)=y_{sp}(t)-y_{m}(t)$$

where e(t) is the error signal, $y_{sp}(t)$ the setpoint, and $y_{m}(t)$ the measured value of the controlled variable. The controller output is proportional to the error signal in Equation (2),

(2) $$p(t)=\overset{‾}{p}+K_{c}\cdot e(t)$$

where p(t) is the controller output, $\overset{‾}{p}(t)$ is the bias steady state value, and $K_{c}$ is the controller gain. The main features of a proportional controller are the ability to adjust the sensitivity of the desired setpoint by adjusting the controller gain, as well as adjusting the sign of the control gain to negative or positive to decrease or increase, respectively, as the error signal increases. The disadvantage of a proportional controller is that a steady-state error occurs after a set-point change or a sustained disturbance that the controller cannot accurately account for. This error will continuously be present but for simple applications may not be an issue. This steady-state error can be eliminated by an integral controller in tandem with the proportional controller. Integral controllers take the form:

(3) $$p(t)=\overset{‾}{p}+\frac{1}{\tau_{I}}\int_{0}^{t}e\left(t^{*}\right)dt^{*}$$

where t is the signal time, and $\tau_{I}$ is the integral time. Integral control will eliminate steady-state offset error by monitoring how p(t) changes over time with respect to e(t). Thus, the integral controller actively works to attain a steady-state error of zero. Therefore, an integral controller is not normally used by itself, as it cannot quickly respond to the immediate error detection like a proportional controller. Many proportional-integral (PI) controllers are thus used for liquid, steam and temperature control within industry as it is simpler in its execution and less noisy than more advanced forms of control. Yet, the PI controller has limitations in its high starting overshoot and sluggish response to sudden disturbances [22]. To account for slow response times a final component can be added known as derivative control. Derivative controllers will act to anticipate the next iteration of the error signal and respond accordingly. By automatically incorporating prediction of an error signal, several systems can be automated based on well tuned set-points and a defined error signal if incorporated with other controllers like the proportional, or integral controllers. In fact, the derivative controller will not be used alone, and will be seen as either a proportional-derivative (PD) controller or a proportional-integral-derivative (PID) controller. Derivative control takes the form in Equation (4):

(4) $$p(t)=\overset{‾}{p}+\tau_{D}\frac{de(t)}{dt}$$

where $\tau_{D}$ is the derivative time. The derivative controller is known to stabilize the control process and improve dynamic response times. The main disadvantage of derivative control is that this controller will react to noisy signals by amplifying the noise from the input signal and try to account for it reducing response times. Thus, for certain applications, a PI controller may be better suited, for example in maintaining flow control. The final summation of each of these controllers can be seen in Figure 6 and is denoted as a PID controller scheme.

A PID controller allows users to take sensor readings from a desired state and produce an output that is calculating the proportional, integral, and derivative summation of the error signal to meet the desired output state. As each component of the PID controller is added, more stability and control of the system is achieved. PI and PID controllers have been the dominant control technique for many decades within large-scale commercial industries, with at least 97% percent of the control system in place utilizing some form of PID control [23]. There are several variations that have been implemented to utilize PID controllers over the years with the most widely utilized being the parallel, series, and expanded forms. Each form has its own advantage based on the application but it is typically observed that when configured with the same derivative filter factor, the series form of the PID controller produces smoother adjustments than the parallel version, at the expense of a slight decrease in best achievable performance [24]. All while, the expanded form is best used for controller tuning and fine parameter adjustments [25].

There are many variations to each of these methods, with some applications taking distinctive approaches to overcome the inherent disadvantages of the PID controller. High frequency sensor noise problems can become severe in some applications, due to the presence of the D component of the PID controller. By altering the controller structure slightly, it is possible to obtain the intended benefits of derivative action, without taking the derivative of the error function [26]. One such example is the Pseudo-derivative feedback (PDF) controller. A PDF controller can be used for unique applications like adjusting the current control of a three-phase grid-connected inverter with LCL filters. The controller helped in significantly improving the transient response of the system [27].

#### 2.2.3. Advanced Process Control—Model Predictive Controller

**Figure 7.**
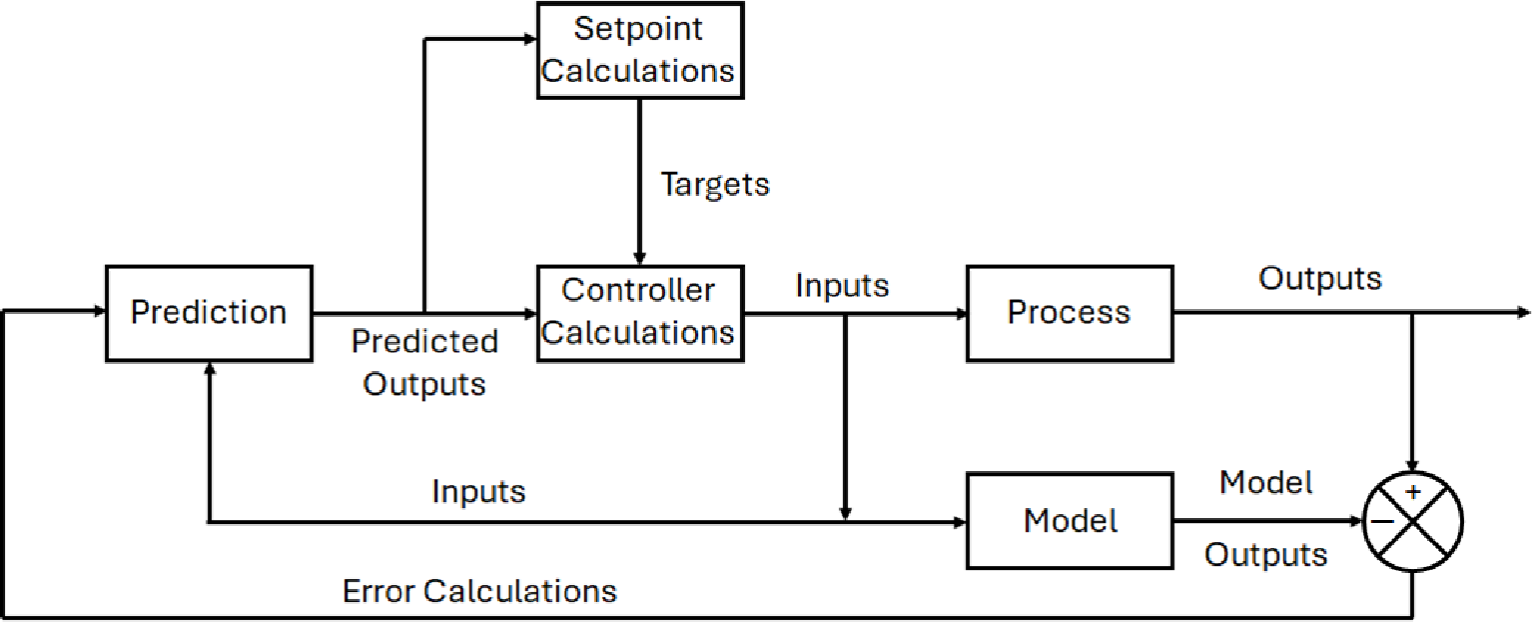
Block diagram control layout of a model predictive controller (MPC) implementation.

The next evolution from the PID controller and its various iterations is what is known as advanced process control (APC). One of the subsets of APC is the model predictive controller (MPC). MPC can be found now in a wide variety of manufacturing environments including power plants, petroleum refinery applications, chemicals, food processing, automotive, and aerospace [28]. MPC uses a system model to predict the future states of the system and generates a control vector that minimizes a certain cost function over the prediction horizon in the presence of disturbances and constraints [21]. The first element of the computed control vector, at any sampling instant, is applied to the system input, and the remainder is discarded [21]. This process of the MPC begins again on the next iteration of the specified time stamp [21]. The advantages of MPC over PID controllers is that: (1) the process model captures the dynamic and static interactions between input, output, and disturbance variables, (2) constraints on inputs and outputs are considered in a systematic manner, (3) the control calculations can be coordinated with the calculation of optimum setpoints, and (4) accurate model predictions can provide early warnings of potential problems [25]. The objective of the MPC control calculations is to determine a sequence of control moves (i.e., manipulated input changes) so that the predicted response moves to the setpoint in an optimal manner [25]. MPC controllers are also particularly well suited for the control problem inherent to plasmas based on the multi-variable nature of system dynamics as well as the need for constraint handling [29]. An example of a basic MPC scheme can be seen in Figure 7 highlighting the path input and output signals take to perform set-point calculations.

Like PID controllers there are different variations that can fit under the definition of an MPC, while still under the APC subset. For example, a nonlinear model predictive controller (NMPC) functions similarly to an MPC controller, just without the requirement for mode linearity. To properly control nonlinear processes, a nonlinear dynamic process model must be used [28]. An NMPC controller can handle processes with models that have varying dead-times and lag-times, and involves the repetitive solution of an optimal control problem at each sampling instant in a receding horizon fashion [30]. NMPC has been postulated as an ideal method for plasma medical therapy due to its effectiveness in handling nonlinear control costs at fast sampling times, while guaranteeing satisfaction of safety-critical system constraints [31]. Unlike linear system identification though, there is no uniform way to parameterize general nonlinear dynamic systems. A type of nonlinear model utilized for NMPCs typically includes artificial neural networks (ANN) [28].

#### 2.2.4. Artificial Neural Networks for Nonlinear Model Predictive Controller

**Figure 8.**
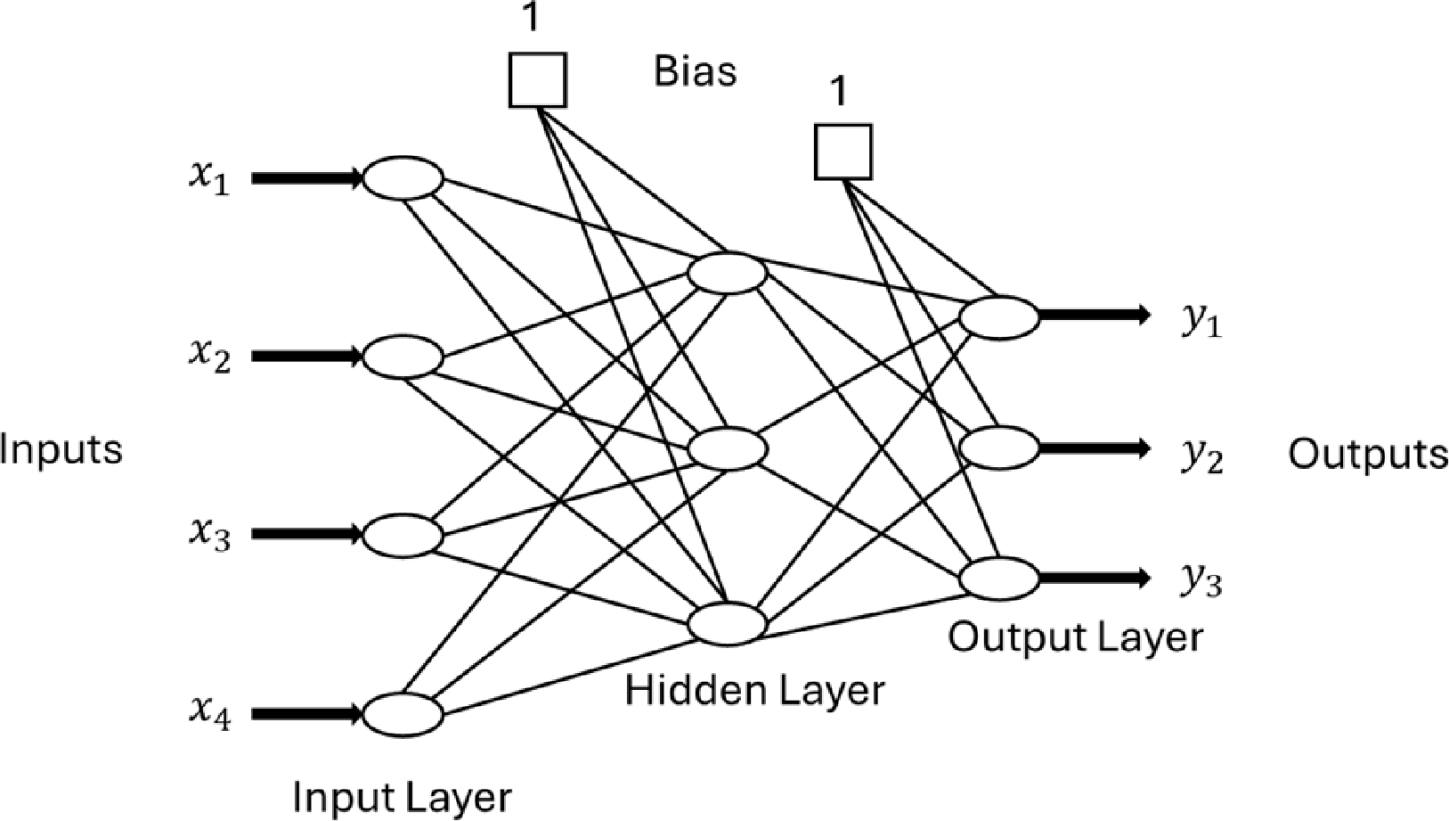
Multi-layer neural network with three layers, ten weighting nodes, and two bias nodes.

Artificial Neural networks are an important aspect to nonlinear models and are currently beginning to be implemented in industrial processes like chemical reactors and even medicine [25,32]. After the success of MPCs, and most recently NMPCs, within the control industry, ANNs have emerged as the next frontier in computational based control mechanisms. For sufficiently computational intensive control schemes like NMPCs, ANNs have been able to provide the constructional framework to make extremely complex control strategies a reality. Functioning in the same vain as the control schemes before, ANNs utilize nodes that are comprised of input signals, outputs, and weighting functions. These nodes are then separated into layers such as an input, hidden, and output layer that adjacently connect to one another. An example of a multi-layer neural network node is shown in Figure 8. The weights for each node are unknown until inputs and outputs are provided, with large non-linear models being comprised of many unknowns. If enough nodes are utilized, an input–output process can be accurately modeled by a neural net model [25]. Once a neural network layout has been established, these models can be trained to actively predict and estimate various parameters for the system being utilized. Like the control schemes mentioned above, these neural networks can have many variations with just one example being a radial basis function neural network (RBFNN). Radial basis function networks are distinguished from other neural networks due to their universal approximation and faster learning speed. An RBF neural network is a type of feed forward neural network composed of three layers, namely the input layer, the hidden layer, and the output layer [33]. Implementation or RBFNNs for plasma endpoint detection for semiconductor fabrication have already been proven viable by trained and tested models [34]. Deep neural networks (DNN) are another form of ANN with more complexity in terms of the amount of nodes and layers. DNN have more recently been employed in plasma medicine research towards identifying a dosage for prospective patients [31].

#### 2.2.5. Reinforcement Learning Control

**Figure 9.**
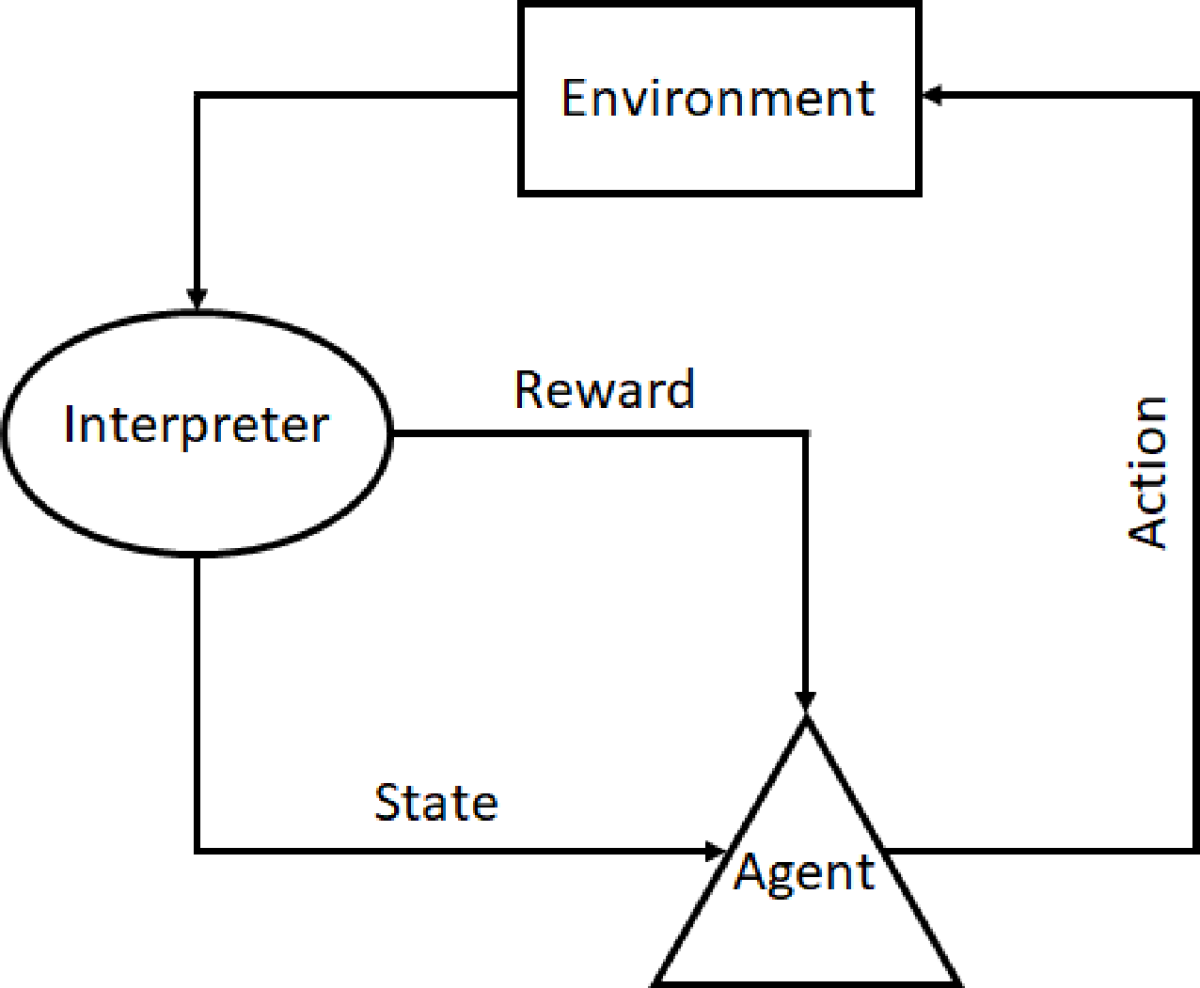
Reinforcement Learning (RL) flow diagram, depicting an agent taking actions from an environment that can be interpreted as a reward or penalty to produce the next state.

Reinforcement learning (RL) is a control strategy originally derived from process control optimization, that was developed on the basis of self-learning, and environmentally driven data models. Upon its original introduction though, it proved less efficient than the, at the time, well established PID control schemes [35]. It would be largely ignored in control theory practices till the onset of developments into DNN and ML. Advancements in computational capabilities in the modern era allowed for elements of the data gathered during RL to be extracted as “features” to be utilized in trained models to make RL a viable, and at times, more efficient advanced form of adaptive learning when working with process control based tasks. RL itself is based off the Markov decision process, which describes the mathematical modeling of a decision-making process using discrete time steps. For each step taken, this results in an action that creates a new environment state. Therefore, the current state is based on the sequence of previous actions taken. RL works by providing a reward to an “agent” when a desired state is achieved. An example of a RL learning scenario is shown in Figure 9. DNN and ML can help in identifying these rewards to correctly produce the next best action to be performed by the designated agent. Thus, an ANN can be trained to take optimal actions to maximize a reward (or minimize a penalty) through continuous feedback during training [36]. Learning-based methods, such as reinforcement learning, do not unambiguously fall in the supervised or unsupervised learning categories. Such methods are generally considered separately, or under the umbrella term semi-supervised learning [37]. Two models can be developed from this system being a model-based or model-free system. Model-based systems are best employed when the environment is well defined and unchanging, while a model-free approach is best implemented for a complex, dynamically changing environment. It is with this understanding that Witman et al. suggest that RL methods hold promise for learning-based control of atmospheric pressure plasma jet (APPJ) applications where the treatment of complex substrates can have time-varying or non-uniform characteristics [36].

With the onset of artificial intelligence (AI) and ANNs, the future of controlling plasmas is promising thanks to the computational efforts that can now be achieved. A new era of control engineering has started with ANNs and RL able to contribute towards NMPCs through trained inputs and outputs. The ability to control nonlinear parameters is beginning to show promise, and the idea of controlling plasma for regular medical applications is soon to be realized.

## 3. Plasma Control

### 3.1. Plasma Control Concepts

With an underlying background on control strategies, it becomes evident that once plasmas need to be controlled, the strategies and parameters needed become increasingly complex once more states of the process start to be considered. Plasma control can take many forms depending on the given application. Two of the most well-established plasma control applications today, are the etching and deposition and fusion industries. These two fields were the first to actively produce control strategies that could manipulate plasma for vastly different purposes. While similarities exist amongst these industries, such as operating at various pressures other than atmosphere, and utilizing magnetic fields to control the density of the plasmas, several differences are obviously present as well. It is with this underlying principle though, that plasma is still the state that is attempting to be controlled. Each of these fields have utilized similar control methodologies to achieve their specific applications and have borrowed strategies from one another throughout their development to the present day. While plasma medicine may have been hypothesized before the other two fields conception [38], much of the strategies and control schemes utilized by the plasma field today have their roots in the pioneered work of these two sub-fields. Therefore, it is of vital importance before reviewing the current state of plasma medicine control, to highlight many of the effective techniques and strategies utilized by other areas of plasma research. It is with this knowledge, that future insights can be gained for prospective medical applications in the future. For example the use of PID controllers to actively manipulate plasma current for shaping of the plasma in fusion, was later utilized by the etching and deposition industry for temperature, and pressure control to manipulate plasma conditions [39].

#### 3.1.1. Etching and Deposition

Plasma etching has been employed for semiconductor processing since the 1960s [40]. As one of the oldest and most successful utilization of plasma in industry, it is worth taking a look at the control and endpoint detection strategies employed by this industry. Most industrial plasma processes are dependant upon the control of plasma properties for repeatable and reliable production. The speed of production and range of properties achieved depend on the degree of control [41]. To achieve better control over the plasma etching processes in semiconductor manufacturing, real-time endpoint detection was introduced in the 1970s [42]. A variety of different sensors and diagnostics have been utilized for endpoint detection and process control. The most commonly used diagnostic has been optical emission spectroscopy (OES), as it is non-invasive and compatible with industrial plasma setups. OES evolved from the monitoring of a single emission line, corresponding to a product or reactant in the process chamber, to a ratio of emission lines to take into account drift over time in the emission signal, to multi-wavelength monitoring [43]. Due to the multivariate nature of OES signals, it is challenging to select the wavelengths that include important endpoint information. Wavelengths are commonly chosen based on prior knowledge on reactants and products, which may omit critical information for endpoint detection and cannot react to process variations [44]. With prior knowledge of the process and the optical signature of normal process conditions and certain common failures (e.g., gas cylinder empty, air leaks, failure of mass flow controller or power generator), a real-time and active control system was designed that constantly compares OES spectra with stored spectra of “normal operation” and a library of spectra of common plasma failures and unique differences for different product types [45]. With the rise of Neural Networks and Machine Learning, patents for employing these strategies in conjunction with optical emission spectroscopy and other process signatures were introduced in the 1990s (e.g., [46,47]. Data-driven wavelength selection and more universally automatic wavelength selection algorithms have continuously improved and were recently reviewed in [44]. In their study, they introduced an endpoint method employing a Gaussian Mixture Model and continuously updated endpoint suggestions to determine the optimal endpoint, allowing to be employed in systems where prior information on wavelength is not available or to account for chamber-to-chamber variations [44]. While the algorithms to make endpoint decisions based on OES signals have continuously improved over the last decades, this method can be insufficient for an active control of plasma densities [48]. Emission intensities are a function of electron density $n_{e}$:

(5) $$I\;\approx \;n_{e}n_{g}K(T_{e})$$

Additionally, they depend on the density of the species g and the rate constant K which is a function of the electron temperature. Thus, the emission intensity can remain constant while the electron density is changing. With electron density, the plasma state and corresponding film properties were observed to change in a deposition process [48]. Woelfel et al. introduce a controller using active plasma resonance Spectroscopy (ARPS) probe diagnostics to measure electron densities in the plasma. The measurement of electron density and/or the energies of electrons and ions gives much more information about the plasma and small changes in the process and may thus be a better parameter for active plasma control. In their study, Woelfel et al. used a plasma-based feedback control system including a feed-forward controller that determines a reference for the measured electron plasma frequency (which is directly related to electron density: $\omega_{\text{p}}=\sqrt{\frac{e^{2}\cdot n_{\text{e}}}{m_{\text{e}}\epsilon_{0}}}$ with respect to the desired frequency and a desired reactive sputtering mode. The control system consists of an estimation unit and a PI controller [48]. However, to measure these quantities, invasive diagnostics are required such as ARPS with examples being the multipole resonance probe (MRP) [49,50] or the hairpin probe [51,52]. Other probe diagnostics, such as the widely used Langmuir probe, are also invasive and come with their own set of problems [53,54]. Another diagnostic used for monitoring plasma processes is a residual gas analyzer (RGA) or mass spectrometry. Many diagnostics and sensors are commercially available and implemented in the semiconductor industry, glass coating industry, and other. As the industries are moving towards intelligent manufacturing, from active decision making towards predictive, cognitive, and self-actuating systems, the level of control of individual plasma systems is a crucial aspect that will inevitably follow the automation demands of intelligent manufacturing.

An example of a diagnostic application of relevance in deposition that is applicable for medical plasma treatments as well is the idea of process control drift. This process is characterized by gradual system aging and persistent environmental drifts that affect the control performance of the plasma deposition process [55]. The problem of process control drift is most relevant in applications where duration times in closed-loop control systems could be affected. Process control drift, or lack of calibration of the closed-loop in question, would be prudent for medical applications as well since CAPs can exhibit sharp spatial gradients in both temperature and reactive species concentration, making them exceptionally sensitive to exogenous disturbances when treating biological substrates. Even slight changes to the target conditions, or to the distance between the plasma source and the target, may result in irreproducible results in otherwise similar experiments [37]. These changes can disturb models developed in-vitro, making them non-transferable from one experiment to the next. Thus, a means of calibration or allocation to the task at hand through automatic calibration would be beneficial. Cho et al. implemented such a process for plasma deposition known as Bayesian optimization (BO) which allows for automatic calibration of advanced optimization- and learning-based controllers within closed-loop control systems [55]. By time varying the Bayesian optimization (TVBO) approach with sequential process runs, Cho et al. tested a new method where each run was represented by an integer index. Each run index could account for the nonlinear or non-stationary dynamics to produce a run-indexed TVBO (RI-TVBO) that could effectively cope with gradual and persistent system drifts. The performance of this system was evaluated in three trial runs that effectively tested the monotonically increasing, decreasing, and continuously changing forms of drift experienced during plasma treatment. Tuning parameters of flow rate, and applied power were adjusted in a kHz-excited APPJ during experimental trials of a thin film deposition process, assisted by an offset-free MPC controller. To highlight the effectiveness of this process: No optimization, BO, TVBO, and RI-TVBO were all compared by the three trials. The trials were compared through the average thin film thickness, optical emission intensity of helium, and maximum surface temperature. Overall the RI-TVBO case outperformed every method, for each trial, given the varying degrees of process drift proposed. By achieving an auto-tuning optimization process for varying degrees of process drift, the potential applications for auto-tuning with RI-TVBO in plasma medical applications are substantial. The need for online, substrate dependent, auto-tuning could help bridge the gap in creating a designated universal plasma treatment methodology for treating various forms of biological substrates that could be encountered during clinical treatments.

#### 3.1.2. Fusion

The pursuit and idea of achieving a net positive fusion reaction for energy applications has lead to the development of varying plasma control mechanisms over the years. The initial idea became pertinent with the onset of the idea that controlled fusion could be utilized to meet humanities ongoing and ever increasing energy demands. After the development of the world’s first nuclear reactor, the Chicago Pile-1, discussions amongst Fermi, Kerst, Landshoff, Teller, and R.R. Wilson at Los Alamos National laboratory deduced that the control of fusion energy depended entirely on understanding plasma physics at the fundamental level [56]. A high density of ions would need to be confined and held together at extremely high temperatures for an ample amount of time. Then, depending on the type of fusion reaction targeted, these parameters of temperature, density, and confinement time produced one of the worlds most intriguing and difficult control theory problems. Early ideas on how to achieve this form of controlled fusion applied this approach by increasing current to allow for Joule heating of the plasma [57]. Once the plasma was heated to extreme conditions mirroring the sun, it could then be confined through magnetic pinching at a defined location. This mechanism is the underlying principle behind much of the controlled fusion research that has taken place since the 1950s [58]. Initial fusion experiments, where confinement was at the forefront, were labeled as z-pinch experiments. Essentially a column of plasma was driven and confined by a current flowing in the z-direction of the plasma that would heat and confine it. These early experiments into controlled fusion saw most control schemes tailored mainly towards current and magnetic field control. Yet these efforts would soon discover the non-linear nature of the plasma and its instabilities created vulnerability to distributions, thus making control of the process increasingly difficult. Different variations in the plasma chamber would advance into the well known toroidal shape, known as the tokamak. Tokamaks proved more efficient in controlling the plasma parameters, increased stability, and could achieve longer confinement times. Though there are several variations and ideas into how to achieve controlled fusion, most of the proposed control schemes in research revolve around implementation of tokamak based reactor designs. Several of the control schemes currently in practice still have a basis in current control, but more advanced forms of current control have been implemented since the 1950s that utilize real-time feedback control. For example the JET tokamak in Europe utilizes feedback control to actively manipulate magnetic fields to confine, shape, elongate, and even change the location to which the plasma is sustained. Today magnetic control is the basis for most of the fusion based control methodologies as it helps in the heating process as well as maintaining the plasma through the reduction of wall interactions that can degrade the chamber and reduce efficiency of the fusion process.

Plasmas are controlled through a magnetic field control system in fusion devices as highlighted by De Tomassi et al. in which the main components in today’s experimental reactors are the “vertical stabilization controller”, and the “plasma shape controller”. Vertical stabilization is usually handled by a feedforward or PID controller method with some variations that may include a ramp-up or ramp-down functionality that would incorporate a double-integral transfer function to control the current through the central solenoid or poloidal field (PF) coils to overall control the magnetic fields. Shape control, on the other hand, is typically handled by an MPC controller to retroactively predict and adjust the plasma position and shape that is needed. The vertical stabilization (VS) system is essential for elongation of the plasma, with the main objective being the counteraction of disturbances, such as Edge Localized Modes (ELMs), and fast disturbances with tokamaks [59]. The system in turn is controlled by manipulating the current in the PF coils as control variables. The current from the PF coils generate a radial magnetic field, which is needed to apply the vertical force used to stop the plasma column and control its vertical stabilization. Current and plasma vertical speed are determined by magnetic diagnostics to supply an adjusted voltage setpoint to alter the magnetic field for real-time magnetic field adjustments. The plasma shape controller on the other hand helps in adjusting the location and shape of the plasma to achieve high efficiency discharges. High efficiency can only be achieved by actively adjusting and compensating for plasma boundary conditions in real-time with the onset of disturbance like plasma wall-gap interactions. The main objective of the plasma shape controller is creating the shape of the last closed flux surface within the vacuum chamber by tracking a set of plasma shape descriptors [59]. These descriptors are tracked and adjusted to meet the desired setpoints based on flux probe diagnostics, and plasma current.

An example of these controllers in practice was highlighted by Degrave et al. in which machine learning control is utilized to manipulate the plasma shape and temperature [60]. Degrave et al. created a tokamak magnetic controller design that autonomously learns to command control coils by utilizing reinforcement learning (RL) to generate a non-linear feedback controller [60]. The proposed design scheme achieves elongation, conventional shapes, complex shapes, and two separate sustained plasma discharges simultaneously within the plasma chamber. The location, current, and shape for these configurations are tracked in the first layer of the control scheme by measuring plasma current, position, frequency, and pressure. The second layer of the control scheme is then implemented through the use of an RL algorithm that collects data from a simulator to find a near-optimal policy. Lastly, the control policy is combined with associated experiment control targets into a compiler tailored towards real-time control [60]. Overall, Degrave et al. demonstrate a new approach to controller implementation that can help in finding a particular controller method or scheme in shaping the plasma and managing the plasma boundary in real-time. By utilizing reinforced machine learning an optimized controller method for managing plasma shape can be worked out in real-time to achieve plasma shape control not otherwise achievable from conventional controller methods designed thus far.

Currently, other controlled outputs like plasma current, plasma resistance, and power, can be adjusted through applied voltage and current to the PF coils within most fusion devices today. The JET fusion reactor has even implemented a real-time central controller which expands the control of the system by manipulating gas flow rate and auxiliary heating of the system [61]. Offline models and online models can be computed and complimented as data-driven models into the control for predictive modelling. Additionally, redundant controllers are added for each control system for reliability and safety. DIII-D is another fusion experiment in the United States that includes real-time feedback control for various parameters like electron temperature, edge stability, disruption, density, and pressure [61]. Density is regulated within DIII-D by CO_2_ interferometers, while equilibrium and edge stability are controlled by the current. Disruptions are managed by adjusting gas flow rates. Several of these parameters are handled by PID controllers and multi-variable state based controllers operating on a feedback control system. Like JET, DIII-D has the capability to run previous test data in dynamic simulations in order to test future control algorithms [61]. Advancements have been proposed to each of the currently established areas of control like vertical stabilization. Sotnikova et al. highlight that MPC control would allow for better performance over traditionally used PID controllers for this application [62]. Vertical stabilization has until now normally been handled by PID controllers due to the computational load of acquired data necessary for management of this task given all the input parameters. PID controllers have been able to respond quicker due to the lack of processing time required to model and predict within the necessary response time. However, the transient response time in a constrained MPC-controller has the ability to produce reliable and fast response times when constrained correctly by optimized weighting functions [62].

### 3.2. Plasma Control Parameters

**Figure 10.**
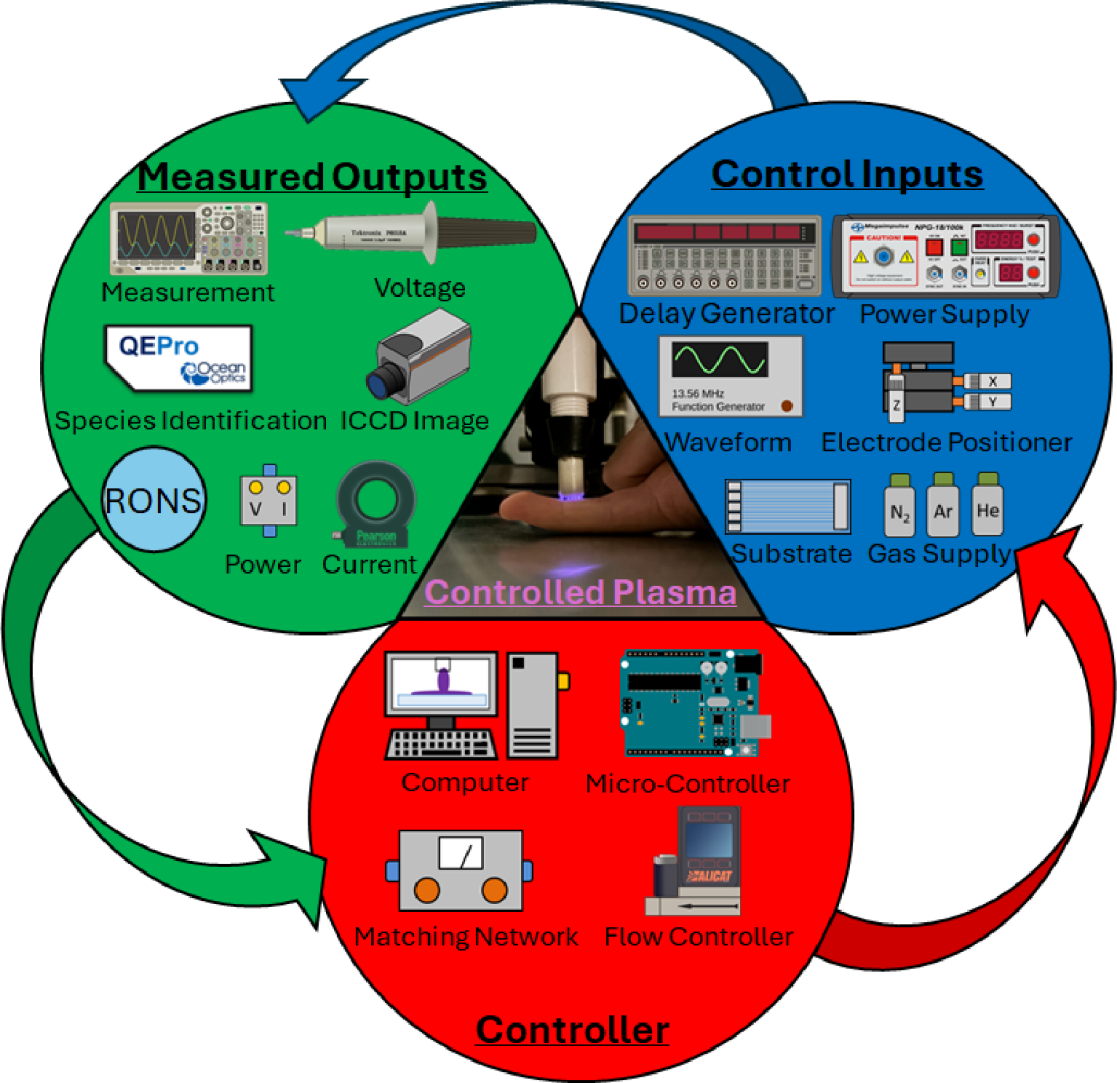
A basic control of plasmas overview depicting plasma inputs that can manipulate plasma parameters (Control Inputs), the measured outputs of the plasma (Measured Outputs), and the controllers that feed directly back the the inputs that need to be adjusted for the desired outputs (Controller).

After highlighting the two areas where plasma control concepts have been established, via semiconductor manufacturing and fusion based applications, we will now take a more detailed approach in describing the various plasma parameters that directly allow for the manipulation of plasmas in this subsection. Different applications can be achieved through the manipulation of parameters like voltage, current, power, flow, signal, and treatment time. Following an explanation on the importance of each parameter, appropriate examples will be presented that highlight areas where applications of the manipulation of these variables have been successful. Additionally, the control method that was utilized to achieve control of the specific parameter will be extrapolated on in an effort to directly tie future applications in plasma medicine. An example of several of the inputs, outputs, and controllers that should be considered in this section, can be seen in Figure 10, illustrating a variety of possible control inputs, outputs that can be measured, and controllers that can be utilized.

#### 3.2.1. Voltage Control

**Figure 11.**
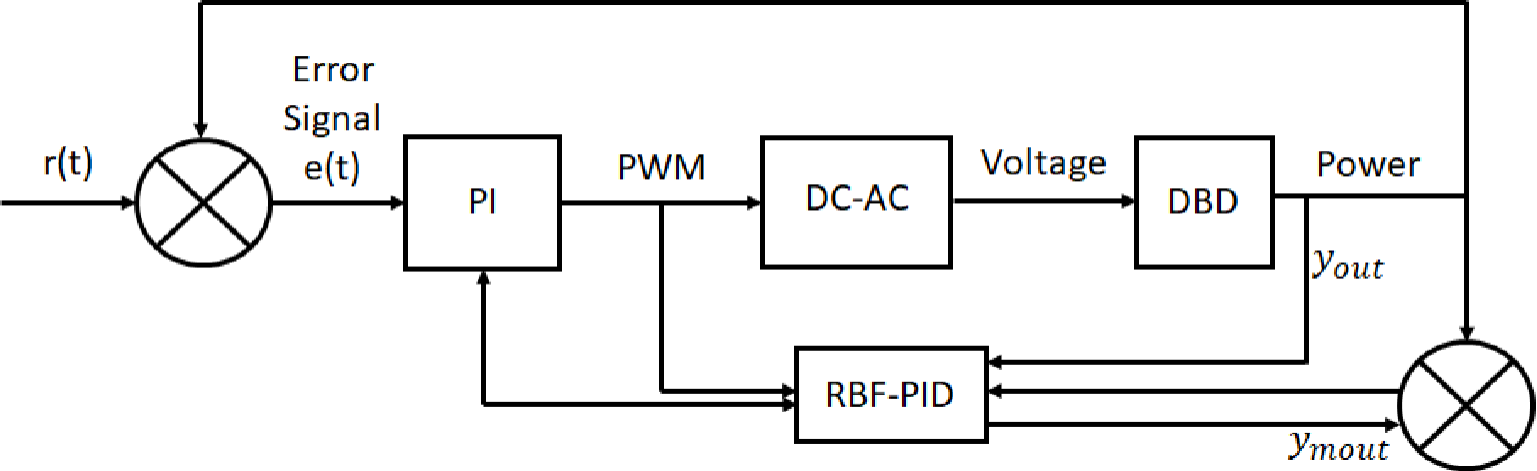
Rendering of a control scheme used for an RBF-PI controller to manipulate input voltage based off predicted power measurements.

Voltage is one of the main driving factors in the ignition of a plasma and the main subsequent control parameter in medical and other plasma based applications. The breakdown voltage needed for the ignition of the plasma is dependent on several factors including chamber, geometry, medium, pressure, and temperature. Voltage control can be manually tailored through the electrode geometry in its thickness, size, and shape, due to electric field enhancements [63]. Voltage control in plasmas can also be achieved through the adjustment of the supply voltage in a power supply. Micro-controllers can be used in tandem with a power supply to adjust the applied voltage based on the desired application in automated applications. An example of this can be seen in a regulated self-tuning power supply based on the plasma’s measured deposited power output in a closed-loop PI controller [64]. Xu et al. combined a PI controller as well as a RBFNN model with a PID controller to achieve a self-regulated voltage power supply (see sketch in Figure 11). Voltage in Xu et al.’s experimental setup is controlled by first providing an output of a set duty cycle as a pulse-width-modulation (PWM) signal. The PWM signal will modify the amplitude of the wave, based on the switching frequency processed by a gate driver, to adjust the output voltage. Additionally, Xu et al. implemented a RBFNN that is trained, and calculates the output power received by the substrate. The data collected by the RBFNN is then processed by a PID controller to provide input back to the original PI controller to adjust the PWM signal. The new PWM signal thus incorporates an output based on the error signal received by the RBF-PID controller. The control scheme described is depicted in Figure 11. From this study, Xu et al. were able to control the device behaving in a nonlinear time-dependent regime. Compared to operation of only the PI controller, the RBF-PID controller gives the voltage control a much shorter overshoot and settling time and is overall more steady for voltage control [64].

#### 3.2.2. Current Control

**Figure 12.**
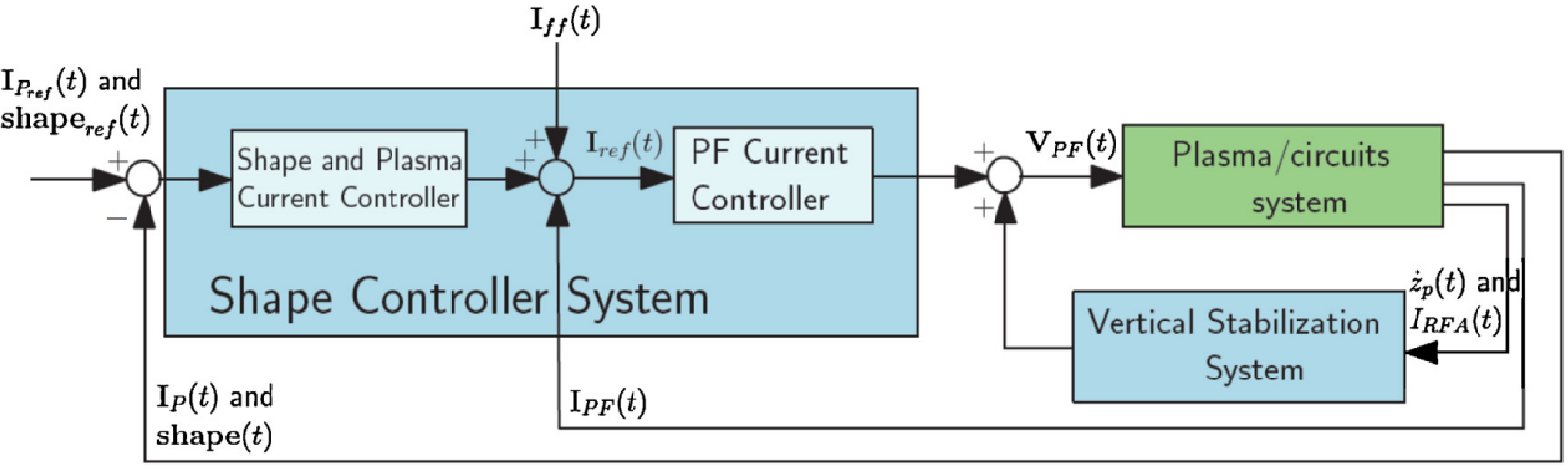
Control block diagram depicting a method for controlling plasma current within the experimental nuclear fusion device JET. Reprinted/adapted from Ref. [65], with permission from, Copyright (2014), Elsevier.

While most atmospheric pressure plasma applications are operated through controlling the voltage, current based control models of plasmas are typically used when certain power requirements are needed for the application such as in high temperature plasma or low-pressure plasma applications. Examples include Ohmic or inductive heating of a plasma in controlled fusion applications. The Joint European Torus (JET) and the International Thermonuclear Experimental Reactor (ITER) use similar plasma current control schemes denoted as the plasma position and current control (PPCC) system [65]. A PPCC system is utilized for controlled fusion experiments in tokamaks where current control takes the form of helping shape and stabilize the plasmas due to the generated magnetic fields, as well as heating the plasma to sustain the temperatures needed to achieve fusion. PF coils surround the tokamak structure and produce magnetic field lines based off the current that is passed through them. These coils help in distributing the plasma current within the system. Current control through the PF coils can influence the strength of the magnetic field containing the plasma. The task of controlling the current is handled by the PF current controller which acts as one of several PI controllers within the PPCC system for JET. The PF current controller is designed to control the current in each PF circuit, as it receives inputs as references for the PF currents. These references are computed as the sum of the plasma shape set-point, and the current required for that shape [65]. An example of this control scheme for the JET PPCC system is shown in Figure 12.

Constant current may be a viable option as well in some plasma applications such as plasma coating or spraying where a set amount of current is needed to produce an electrolytic oxide layer on a specific alloy [66]. Constant current control may be beneficial for faster etching rates but can also run the risk of causing deformations or unwanted damage to wafers during etching if not controlled correctly [67]. In terms of industrial processes, Beck found that plasma anodization was more efficient for current controlled modes thanks to inherent disadvantages that a constant voltage would provide [39]. It was found that: (1) constant voltage had a higher sensitivity to growth kinetics, (2) rapid oxide growth a the beginning of the process damaging the formation of very thin films, (3) growth of oxide films is less controlled due to variability in plasma conditions upon start-up, and (4) the electric field changes considerably creating poorer electrophysical properties [39]. Whether constant voltage control or current control is best depends on the plasma application. Several studies have showcased the advantages of constant voltage and constant current for different plasma applications [68–70]. For medical applications a constant voltage is normally utilized, and has been shown to reduce the amount of leakage current directed to the patient when utilizing an APPJ device with a stationary treatment distance [70]. Additionally, constant voltage has been shown to contribute to higher concentrations of reactive species production [70]. Based on these experimental examples, further investigations into plasma current control schemes could be pursued. Dynamically adjusting the plasma current in response to substrate changes where impedance or reactive species detection is taking place could lead to distinct differences in voltage controlled results for medical applications. Safety tolerances would need to be more strictly monitored but constant current could be a potential control method not initially thought to be investigated for medical applications.

#### 3.2.3. Power Control

Power control can take the form of either manipulating the voltage or current input of the system by having a set power output from the power supply that can be regulated. Power control is often seen in plasma etching applications where the power that the plasma is delivering to the substrate is of high importance since this could influence the shape and depth of the etched features of the wafer [71]. Medical or biological applications require a certain amount of care in the amount of plasma power dissipated to a substrate, since gas temperature will increase with added power, or when introducing a conductive substrate. A threshold or balance in plasma power is crucial since gas temperature has to be below 42 °C to prevent cell destruction when treating a biological substrate [72]. Appropriate plasma power is a key parameter though in generating enough reactive species for inactivation of bacteria [73], wound healing, or cancer treatment [74]. Therefore a balance is needed between the amount of power while still producing the correct amount of reactive species needed for the targeted application.

**Figure 13.**
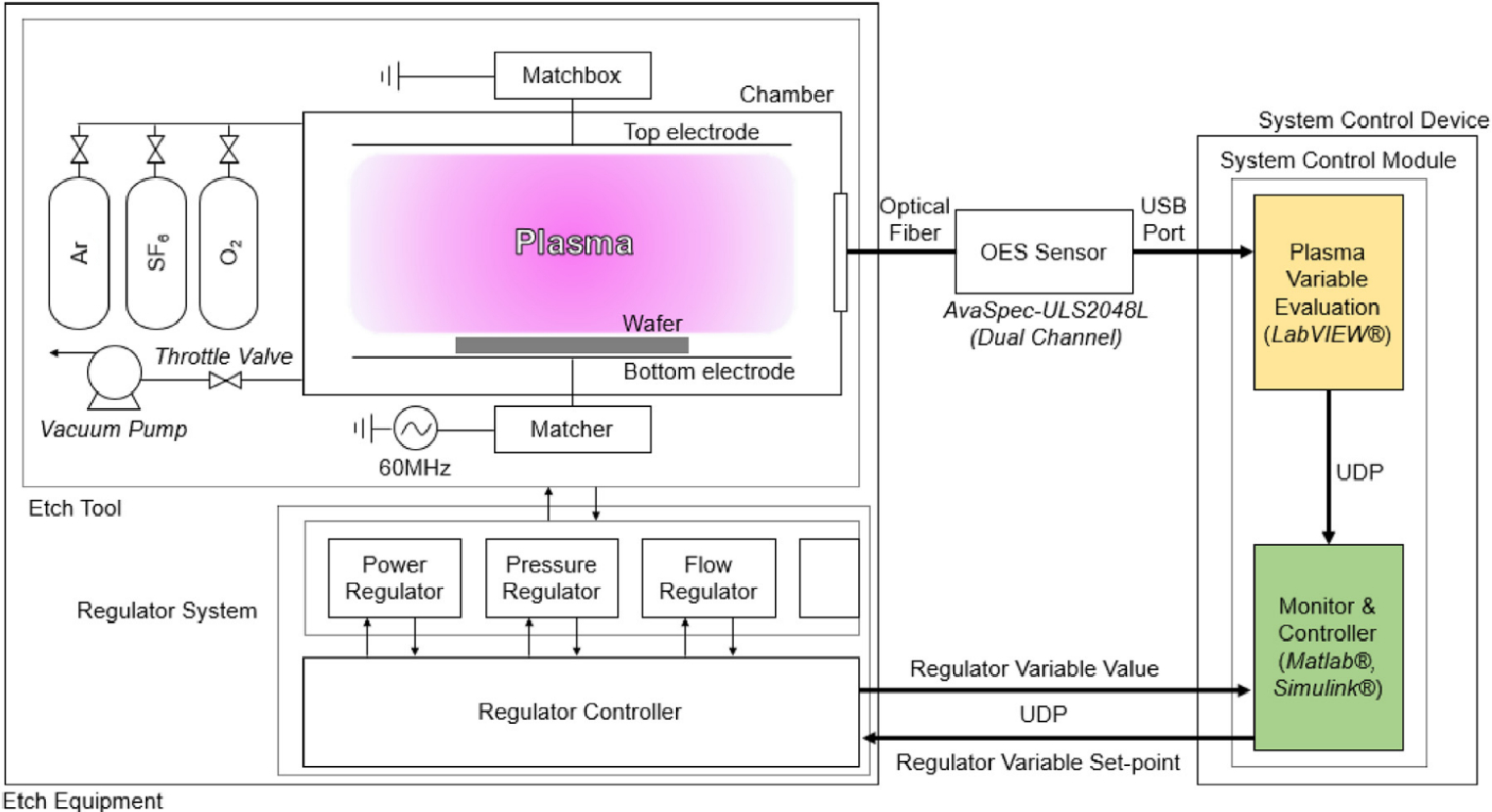
Schematic of CCP etch reactor employed for the use in comparing AMPC and MPC control strategies by manipulating input variables of power, pressure, and flow. Reprinted/adapted from Ref. [75], with permission from, Copyright (2019), Elsevier.

An example of power control can be seen in Figure 13 where electron density set-points are achieved by controlling the power, pressure, and flow in a capacitively-coupled plasma (CCP) etch reactor [75]. The plasma variables of electron density, ion density, electron temperature, ion flux, plasma potential, and electron energy distribution function were measured to help in reaching an electron density setpoint [75]. Radio frequency (RF) power, as well as the other input parameters, were retroactively adjusted through the use of these measured values in real time [75]. Measured values were then processed by the controller to determine a regulator variable value. The regulator variable value was then processed by the regulator controller to distribute output set-points for the power, pressure, and flow. To achieve this level of control, Koo et al. became the first group to use an adaptive MPC (AMPC) control method for a plasma-based system. AMPCs utilize a linear parameter varying method with an MPC controller to achieve a self-tuning strategy based on the linear approximation between the predicted output and the MPC tuning parameters [75]. Overall, the self-tuning AMPC controller displayed 21% better performance compared to a conventional MPC controller for the real-time power control at the sample time level [75].

#### 3.2.4. Flow Control

Gas flow, or fuel for the plasma, is an essential component of many plasma control schemes. Supplying a specific type of gas at a given flow rate can help regulate the properties and effects the plasma will have. If a designated gas type is not utilized, the ambient environment or airflow can be used given the breakdown voltage to ignite the plasma in that medium is satisfied. Flow control as a control parameter is vitally important to industries which involve the manufacturing or coating of structures. Known as thermal spraying or plasma spray technology, this development has provided numerous advantageous innovations to material science and engineering within the automotive, computer, and telecommunications industries [76]. Plasma surface coating requires a precise control of the process gas in order to obtain the desired outcomes for the given material. Plasma treated materials with the correct combination of gas composition and flow rate have demonstrated an increased resistance to temperature, corrosion and wear, [77], and can minimize fluid erosion and abrasive wear of drill bits [78]. At the same time, biomedical engineers have found that with adequate flow control of plasmas, thick layers of bio-adhesive materials can be attached to bio-implants for use by patients [79].

Different control schemes and implementations have surfaced on how to best utilize the outputs of this process to regulate the control of the plasma. Different configurations and applications play a part into these control schemes. For medical applications several devices have emerged that utilize the flow of a gas or flow of ambient air to produce a plasma jet for uses in post-surgical cancer treatment [80], wound healing [74], and the inactivation of bacteria [81]. Each investigation has highlighted the need for precise flow control due to surface temperature fluctuations, and differing modes of operation being in either a laminar or turbulent flow. Flow control of a plasma device is normally handled through a mass flow controller (MFC) and can be manipulated manually or through external control via outputs of the plasma process. A multichannel reactive plasma gas control system was presented by Bellido-González et al., where the controller utilized plasma emission monitoring and target voltage as the input into a high-speed control algorithm for gas input [41]. The control algorithm and parameters were tuned to different process requirements in order to optimize response times [41]. To achieve this process control, Bellido-González et al. utilized a pseudo-derivative feedback controller (PDF). A PDF controller approach was undertaken so that a separation could be established between the measured voltage and optical emission intensity to allow for optimization of each finite element. Exhaust gas was utilized to measure gas partial pressure, while substrate transport coating was used to measure transparency, refractive index and conductivity [41]. Through each of these output elements, Bellido-González et al. were able to achieve an actuation time for gas injection within a range of 10 ms. Up to 4 channels of MFC actuation were achieved to hit targeted voltage thresholds within 5 s of the gas switch being activated [41]. Additionally it was shown that by introducing a second order controlled system, the PDF controller could introduce higher levels of stability to the plasma process of the desired substrate. Gas flow rates responded accordingly with changes to the voltage and optical emission intensities when the plasma passed over a glass substrate. The high speed of control and stability of the controller allowed for increased rates of deposition.

#### 3.2.5. Frequency/External Signal Control

Frequency is another important input parameter that can be adjusted in plasmas through an applied external signal. Low-temperature radio frequency plasmas are essential in various sectors of advanced technology, from micro-engineering to spacecraft propulsion systems and efficient sources of light [82]. Plasma reactors used for etching are often driven at frequencies between 1 MHz and 200 MHz, within the radio-frequency (RF) domain [82]. In particular, 13.56 MHz and its harmonics are popular choices in the etching industry and for medical applications [82]. Control of applied signals can be done in various methods to control different aspects of a plasma. Arbitrary waveform generators can allow for manipulation of an applied signal such as increasing or decreasing the duty cycle, frequency, shape, and amplitude of the applied signal. In some power supplies, by applying an external signal where the duty cycle is manipulated, the overall amount of deposited power can be adjusted [83].

Several examples have emerged in recent years on regulated control of the frequency to tailor towards the substrate that is being treated. Neretti et al. showcased a real time analysis of a closed-loop control system to determine the optimal operating frequency based on the resonance of the circuit [84]. A DBD plasma source equipped with on-board diagnostics was used to measure the output voltage and the charge delivered to the load [84]. Once a load is connected to the high-voltage terminals, a self-tuning procedure is carried out to obtain the best operating frequency based on the impedance of the circuit. Frequency is controlled by an Arduino DUE micro-controller in which a PWM signal regulates the switching frequency of two MOSFETs. Neretti et al. determined optimal frequency by plotting generator efficiency as a function of the switching frequency and discovered that before the circuit hits resonance the generator efficacy increased. Yet, once resonance frequency was reached an increase in current occurred that decreased generator efficiency, providing an optimal operating regime for increased plasma efficacy and control as well as reduced stress to electrical components. An optimal operating regime could be determined based on the dissipated power and switching rate of the MOSFETs that could be self-tuned in an automated process.

#### 3.2.6. Treatment Time Control

The time at which the plasma is ignited and duration of treatment is another important factor to consider for any plasma based application. The length of the plasma treatment can affect the etching and deposition rate and quality of the wafers being created for semiconductor manufacturing. Plasma medicine applications require great care in the amount of time a treatment is to occur. For example, thermal stress on patients has been shown to induce growth inhibition or cell death when thermal effects are too high [85]. Under just the right treatment conditions and time interval Brehmer et al. showcased the reduction of the bacterial load in chronic wounds by a DBD device without harmful side effects [10]. Therefore, it is evident that the correct amount of plasma treatment in either a direct or indirect method is vital towards achieving the positive effects of plasma medicine applications.

**Figure 14.**
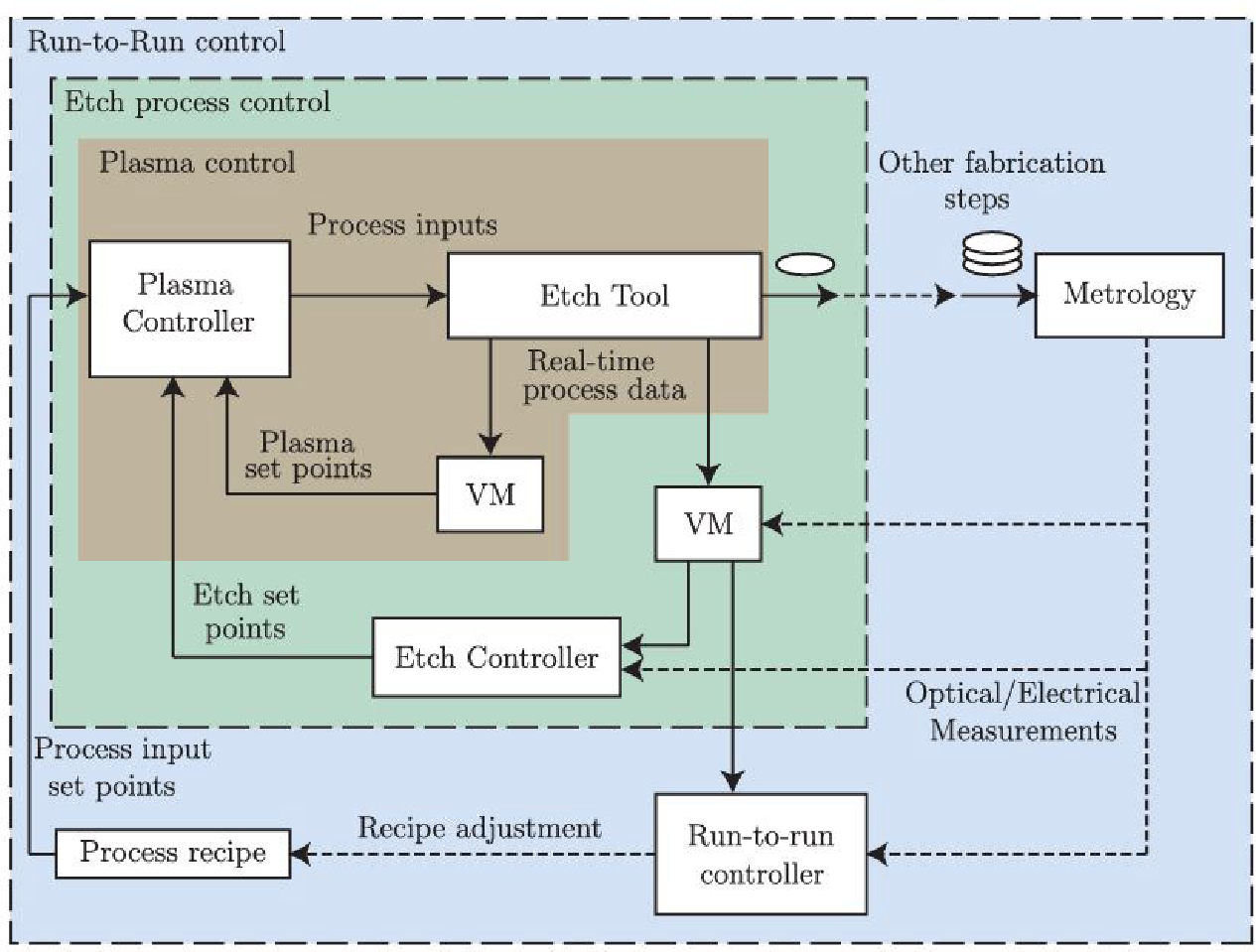
Control architecture diagram depicted the implementation of virtual metrology models and controllers for controlling etch rate time in a CCP reactor. Reprinted/adapted from Ref. [86], with permission from, Copyright (2012), Elsevier.

An example of utilizing time based control of a plasma process is in plasma etching where Lynn et al. showed that for a CCP reactor varying input parameters could be manipulated to optimize the etching process to hit desired etch rates. An advanced process control (APC) scheme was used in order to predict the metrology affects of plasma interactions with different substrates. Metrology effects were cataloged and predicted based off data collected over an experimental range for the device. The data that was collected to determine etch rate was modelled with multiple linear regression (MLR), a back-propagation artificial network, and a Gaussian process regression. The modelled databases predictive functions were then carried out in practice by an MPC controller strategy to actively adjust and manipulate inputs. The controller could adjust applied power, ground impedance, and pressure to achieve a desired etch rate time [86]. The implementation of this control scheme is depicted in Figure 14. Outputs like electron density helped in determining the applied power to the system and were measured by a hairpin resonator probe. A Plasma impedance monitor provided feedback that could adjust a matching network connected to the grounding leg of the reactor to adjust the ground impedance of the system. Lastly, the chamber pressure was adjusted by a gate valve between the chamber and vacuum turbo pump, with a chamber pressure gauge. To handle each of these inputs and outputs, the MPC references the metrology database to give feedback to an etch controller and a run-to-run controller. The etch controller help define the etch setpoints through the adjustment of the ground impedance a specified location, which the run-to-run controller provides the plasma “recipe” of power and flow rate that needs to be achieved by the plasma to reach the desired etch rate target. These values can be simulated offline to calculate a predicted etch rate per second for a set of input variables with a given range. Lynn et al., like others, determined through comparison of two different controller methods PI control was not adequate for handling transients within the startup process of the system. Lynn et al. also discovered inherent weaknesses in using a trained modelling method. The process drift during the etching process would make the models invalid over time, and would require a consistent refreshing of the modelling database in real-time along with onboard diagnostic fed to the metrology model that were too fast to compute. Overall, Lynn et al. were able to provide their control system with each of the input parameters that could predict an estimated etch rate per second. Experimental results showcased a controlled etch rate within 1% of the desired time point was achieved and provided a new method of time control of plasma treatment based on predicted models and real-time diagnostics of the plasma process [86].

## 4. Plasma Control in Medicine

### 4.1. A Brief History of Plasma Control in Medicine

Controlled plasma as a means for medical applications was first hypothesized as early as the late 19th century before the onset of etching, deposition or fusion based applications. It could be argued that plasma control for medical applications began in the 1890s with Nikola Tesla [38]. Tesla showcased that by controlling plasma current through pulsed oscillations on the order of milliseconds the spark gap between electrodes would produce a damped sinusoidal waveform. These high frequency pulsed oscillations (500 kHz–1 MHz) at large voltages ranging from 5–500 kV were found to be harmless and of no discomfort to a person when passing through the human body. Other scientists of the era such as Elihu Tomson would go on to showcase such feats at the 1893 World’s Fair. These early revelations towards the application of controlling high frequency currents in plasmas showcased an increased oxidation and hemoglobin [87], increased blood supply to an applied area [88], antimicrobial effects [89], and lastly an increased immune response before plasma applications were generally understood. These observations would go on to be confirmed through modern scientific approaches by Collet et al. [90], Heuer et al. [91], Laroussi [92], and Miller et al. [93] respectively for each early observation of controlled plasma applications. Yet, even with these early ideas into controlling plasma for its medical applications, pharmaceutical approaches took the forefront of scientific application as it was a better understood form of medicine at the time that could be verified. Additionally, technology of the era could not accurately showcase, or verify, the mechanisms behind the responses seen by plasma human interactions. Thus, controlled plasma therapeutics fell to the wayside until a renewed interest was sparked by increased capabilities in microelectronics that allowed for easier development and testing of such devices to prove their efficacy in medical trials. The first major medical plasma application came in the form of argon plasma coagulation in the 1970s [94]. Argon plasma coagulation is an application of argon plasma discharges in electrosurgery, which is increasingly used in endoscopy. The major application fields are hemostasis, tissue devitalizing and reduction of tissue [95]. APC has superficial thermal effects on tissue in a non-contact manner and has become increasingly popular in coagulation medical applications [95]. With the onset of plasma coagulation becoming successful, an increased interest in cold plasma for medical applications once again surfaced. With increased interest, and several innovations on the development of electrodes and power supplies, clinical trials with CAP devices would soon begin in Europe in the 2010s [10,11].

### 4.2. Plasma Medicine Control Review

Medical plasma applications have long sought to understand the correlation between plasma treatment parameters and the varying effects that are seen through applications like wound healing, cancer treatment, and inactivation of bacteria [92,96,97]. The variation in possible control parameters across the plasma industry, as was demonstrated in Section 3.2, has led the medical plasma community to postulate which of these variables are vital towards effective treatment given the challenges associated with treating biological substrates. Additionally, defining which control method is best suited for handling the non-linear aspects of the plasma, and its reactions, remains an ongoing challenge and point of experimental interest today. Several of these control challenges become increasingly apparent in experimental practice due to the changing cocktail of reactive species produced upon the introduction of varying substrates. This is evident in the actively changing interactions that can occur chemically through enzymic or antioxidant reactions, when plasma interacts with living cells [4].

Evaluating control techniques that played an instrumental role in the semiconductor and the controlled fusion disciplines can potentially provide insights into potential paths to overcome some of the challenges currently facing the implementation of universally defined plasma dose therapies. The field of plasma medicine, since its conception, has utilized many of the same control concepts pioneered by the aforementioned industries in order to achieve similar innovations. For example, plasma species detection was originally used as a means to ensure the efficacy of wafer etching to a desired substrate [98,99]. Yet few examples in plasma medicine are incorporating real-time sensing capabilities in biomedical CAP devices, as sensor capabilities are often limited in the detection of plasma biological substrate interactions. Additionally plasma treatment sensing can be drastically different across experiments due to the intrinsic plasma and surface variability during the treatment.

Early evaluations though by Gjika et al. have proposed the comparison of input parameters towards the overall effectiveness of the targeted application for medical applications with sensor feedback. Gjika et al. proposed the future use of a monitoring control system of cellular responses based on CAP treatment in a continuous read method, with RealTime-Glo assays serving as part of the overall feedback system [100]. By tracking measured power and exposure time as inputs and hydrogen peroxide and nitrite detection as outputs, the overall effectiveness of CAP treatment on cell viability of cancer cells was established [100]. In mapping the effectiveness of each parameter, it was determined that controlling treatment duration and voltage was vital for any future plasma medical device in an effort to suppress cancer cells [100]. It is with this underlying understanding that a variation of certain parameters, like treatment time, can suppress cancer cell viability, that has led the plasma medicine field to strive for a control scheme that can account for output measurements and retroactively change input parameters to achieve the desired aims of a specific task like cancer treatment. Therefore, one of the first approach’s began in PI control.

Plasma medicine can draw parallels to the use of PI controls for plasmas from the successes displayed in fusion applications, where they were first implemented to actively maintain plasma temperature within tokamaks [101]. Similarly, plasma medicine today is expanding and innovating on these early approaches by using PID [102] and MPC [103] controllers to maintain temperature and deposited power for biological substrates. The concept of utilizing reinforcement learning to achieve more flexibility and control of plasmas was also employed to unprecedented success in fusion applications once trained on a simulator model [60]. Plasma medicine applications are beginning to take similar approaches in exploring how previously ignored control methods, like reinforcement learning, could hold promise in actively regulating temperature effects to differing substrates [36]. Properly trained models with reinforcement learning, akin to how fusion based models were trained off simulated ones, show promise in developing specified plasma therapy treatments models in the future as well [104].

#### 4.2.1. Substrate/Target Detection

For plasma medicine applications, the interaction that takes place between the substrate and the plasma is one of the most important aspects to be brought into consideration when evaluating the control scheme to be utilized. The interaction and the species produced directly depend on the type of substrate being treated. Once a substrate is introduced, the parameters of the plasma itself will change. Thus, characterizing how plasmas will behave, with a particular substrate, will be vital towards understanding how this knowledge can be employed in medical applications. The etching industry, currently, already makes use of designated control methods based on the type of substrate used to control the uniformity, yield, and quality of the process [71].

For medical applications the plasma dose delivered to the substrate can vary significantly, particularly when the substrate is electrically coupled to the discharge as can sometimes be the case with DBDs and APPJs [6,105]. Variations in the conductivity and permittivity of biological or liquid substrate should be considered as the impedance of the circuit will be impacted and will differ across experiments. Consequently dissipated power by a plasma device will also be dependent on the impedance of the substrate. Therefore, Hofmann et al. focused on applying in situ measurements of plasma dissipated power and gas temperature when in contact with different substrates, i.e., water, glass, and aluminum [106]. Optical emission spectroscopy (OES), intensified charge couple device (ICCD) imaging, and time resolved power measurements are a few methods that can help in providing insight into how plasma discharges can be altered by different substrates. Hofmann et al. showed a significant increase of both, the dissipated power and gas temperature, when the plasma interacted with water and aluminum. Dielectric treated material, like glass, showed no significant increase in power or gas temperature. It was shown that an increase of power is only starting the moment the plasma touches the conductive substrate. The increase of power was attributed to a change of the equivalent electrical circuit, leading to a more favorable matching between the power supply and the plasma source.

Monitoring plasma impedance is another avenue that has appeared to quantify the effects a substrate has on the total equivalent circuit of the system that could be utilized in plasma medicine control applications. Dubreuil et al. utilized plasma impedance monitoring (PIM) when interacting with different substrates to determine real time endpoint detection during plasma etching of structured bulk materials like Si, Ge, SiC, diamond, and GaAs [107]. High accuracy was achieved by tracking harmonic changes during the etching process which identified the mass differences between ions and electrons leading to real time diagnostics of the plasma state during and after treatment [107]. Substrate emission control is another method that utilizes the substrate to provide real-time feedback control to the plasma process as it interacts with a substrate. The plasma emission feedback system (PCU) was utilized by Ohno et al. to deposit photo-catalytic TiO_2_ films by reactive magnetron sputtering with uni-polar pulsing [108]. Within this study an optical emission detector (OED) was utilized to determine the plasma emission intensity during sputtering. The PCU based off certain emission intensity thresholds for the substrate being used would actively control the flow of oxygen to the reactor chamber to achieve a deposition rate of over 30 nm/min [108]. By actively monitoring plasma emissions during medical treatments, short-lived species only active during treatment of a particular substrate, could be identified.

**Figure 15.**
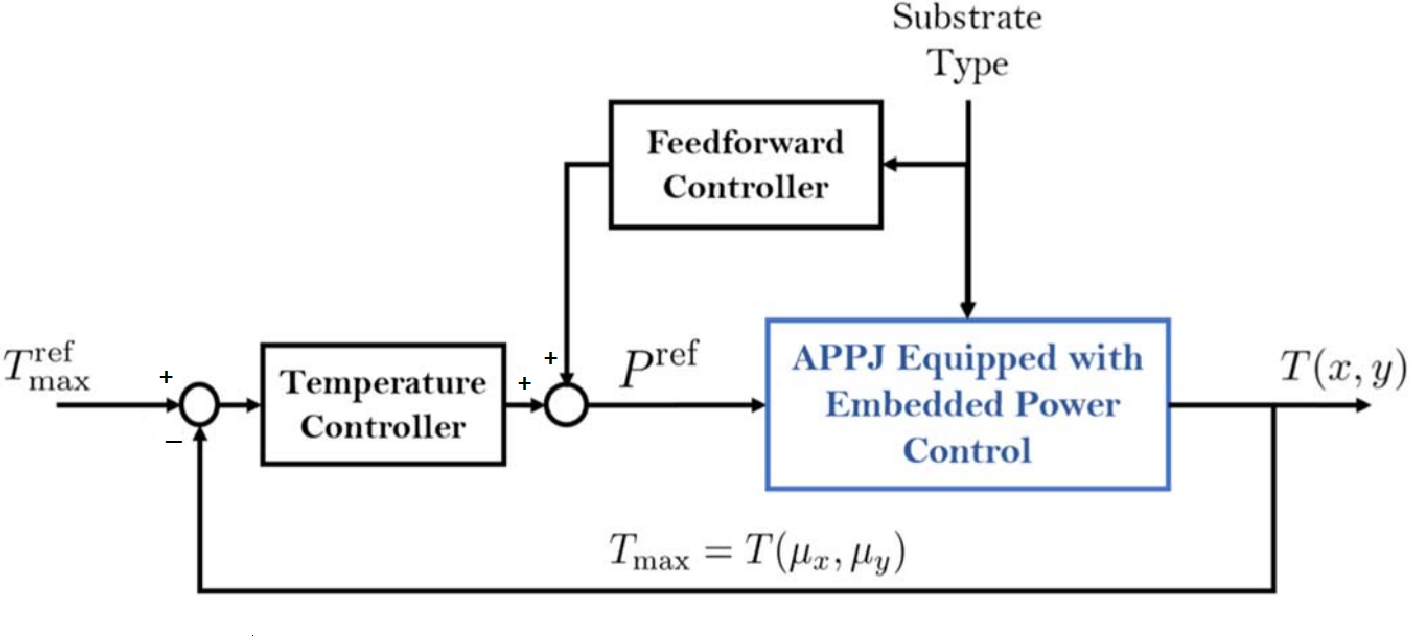
Block control diagram depicting a feedforward controller accounting for substrate type when measuring substrate temperature. Reprinted/adapted from Ref. [105], with permission from, Copyright (2019), IOP Publishing Ltd.

To map the use of APPJs for use in medical applications Gidon et al. implemented the use of a transitional platform that held the substrate and could be manipulated in the x-y direction. This movement of the substrate was done to mimic the use of an APPJ in clinical practice and regulate the spatial thermal dose delivery to a surface [105]. The small surface area coverage of APPJs often requires the translation of the device across a target substrate, which introduces an additional source of disturbance to reliable and spatially uniform dose delivery [105]. By utilizing an MPC controller, Gidon et al. were able to retroactively control the applied voltage, gas flow, and substrate to optimize dose delivery of an APPJ on complex surfaces for use in medical applications. The substrate was actuated in both, the x- and y-direction to account for treatment distance and overall coverage of the surface being treated. These manipulated variables were controlled by an Arduino UNO micro-controller receiving inputs from voltage, current, thermal imaging, and optical emission spectroscopy (OES). For substrate detection in particular Gidon et al. implemented a feedforward scheme into the control layer that would account for the variations and disturbances that arise when transitioning to a substrate that is more (metal) or less (glass) conductive. The feedforward control system was implemented into this design since the role of feedforward controller is utilizing some form of input or sensor to detect a disturbance in the original input signal. Once the feedforward controller has received a specific signal or task, it can detect a variation in the input and then account for it. In the case of Gidon et al.’s experiment, the disturbance to be accounted for was changing from a metal to glass substrate or vice versa, and a preset input setting could be used to allow for more or less power deposition to reach a certain temperature threshold during treatment. Since metal is a more conductive surface, applied power would need to be reduced to maintain the same temperature setpoint. To accomplish the identification of the substrate in real-time, clustering of measured values for OES intensities provided values on the two types of substrates to be deduced. To spatially distribute the thermal dose metric of Cumulative Equivalent Minutes (*CEM*_*T*_) to a substrate, Gidon et al. first solved an offline optimized path for the substrate being treated that would cover 1 cm^2^ per 0.25 min [105] for a defined surface area. The optimized translation trajectory was determined offline in an effort to accurately provide the specified thermal dose uniformly within the designated time frame across the substrate. Real-time diagnostics sent to the MPC controller would then allow for the delivered temperature to the substrate to be retroactively adjusted during treatment to the specified thermal setpoint across the predetermined trajectory. Temperature control was maintained through the manipulation of the flow rate and applied power. An example of the feed-forward control layer is depicted in Figure 15.

Another instance of substrate detection was recently introduced by Behmani et al. where different substrates of varying permittivity and conductivity were treated. The different substrates used (copper, silicon, goat-skin, quartz, and Teflon) had a noticeable effect on plasma parameters such as floating potential [109]. Floating potential and fluctuating electric field were measured with the use of three oriented pin probes. The fast-Fourier-transform (FFT) of the floating potential signal was acquired to determine the change in frequency when the plasma jet was in contact with different substrates. From each of the treated samples it was determined that even though the signal was frequency locked, spikes in the floating potential would create fluctuations in the applied frequency to the substrate. Fluctuations in the floating potential and frequency were found to correspond with streamer propagation and overall plasma activity. Behmani et al. observed that the plasma ignites at a lower applied voltage for higher permittivity and conductivity substrates like copper, while lower permittivity and conductivity substrates like Teflon required higher ignition voltage. The authors suggested that fluctuation signals could provide a good means of input signaling to a feedback controller during real-time diagnostic of the plasma treatment process to control RONS production and delivery and to use variable capacitors or resistors in the ground leg to tune these fluctuations for possible medical applications in the future [109].

#### 4.2.2. PI Derived Plasma Control

Gidon et al. compared the performance of a closed-loop proportional-integral (PI) control system to an MPC control system in simulated cases for an APPJ system. Following the suggestions of Gjika et al. voltage and flow velocity were used as manipulated inputs, while substrate surface temperature, plasma current, and power were measured outputs. Two cases were studied, with the first being the disturbance rejection setpoint tracking of the PI and MPC controller. The second test case studied whether a set dose delivery could be achieved for a stationary APPJ. Gidon et al. proposed a means for accounting for effective thermal dose delivery to a target by utilizing an established parameter known as the cumulative equivalent minutes (CEM) temperature dose metric (CEM_*T*_) for an APPJ [102]. This metric was originally derived based on the dependence of cell death from a medium temperature of 43 °C [110]. The cumulative non-linear nature of plasma effects were considered in an additional, non-thermal dose metric (CEM_*P*_), taking into account non-thermal energy transferred to the substrate. For the first test case the objective was to maintain substrate temperature (≤316 K), plasma current (≤2.5 mA), and plasma power (≤10 W), by manipulating voltage (100–700 V) and flow rate (8–35 m/s) for a treatment duration of 150 s. In order to achieve the regulation of the input variables voltage and flow rate, a PI and MPC control scheme were utilized to compare response and control that could be achieved for each. Gidon et al.’s simulation results were able to demonstrate that the MPC controller, for the first test case, was able to maintain the substrate temperature and voltage and current requirements within the 150 s treatment while exposed to disturbances like separation distance, and varying surface impedances. Few adjustments were made to the input voltage or flow rate. In comparison, the PI controller had overstepped the boundary for both substrate temperature and current while fluctuations in the flow rate were extreme during ignition of the device and through the treatment period to compensate for the violation of the defined constraints.

**Figure 16.**
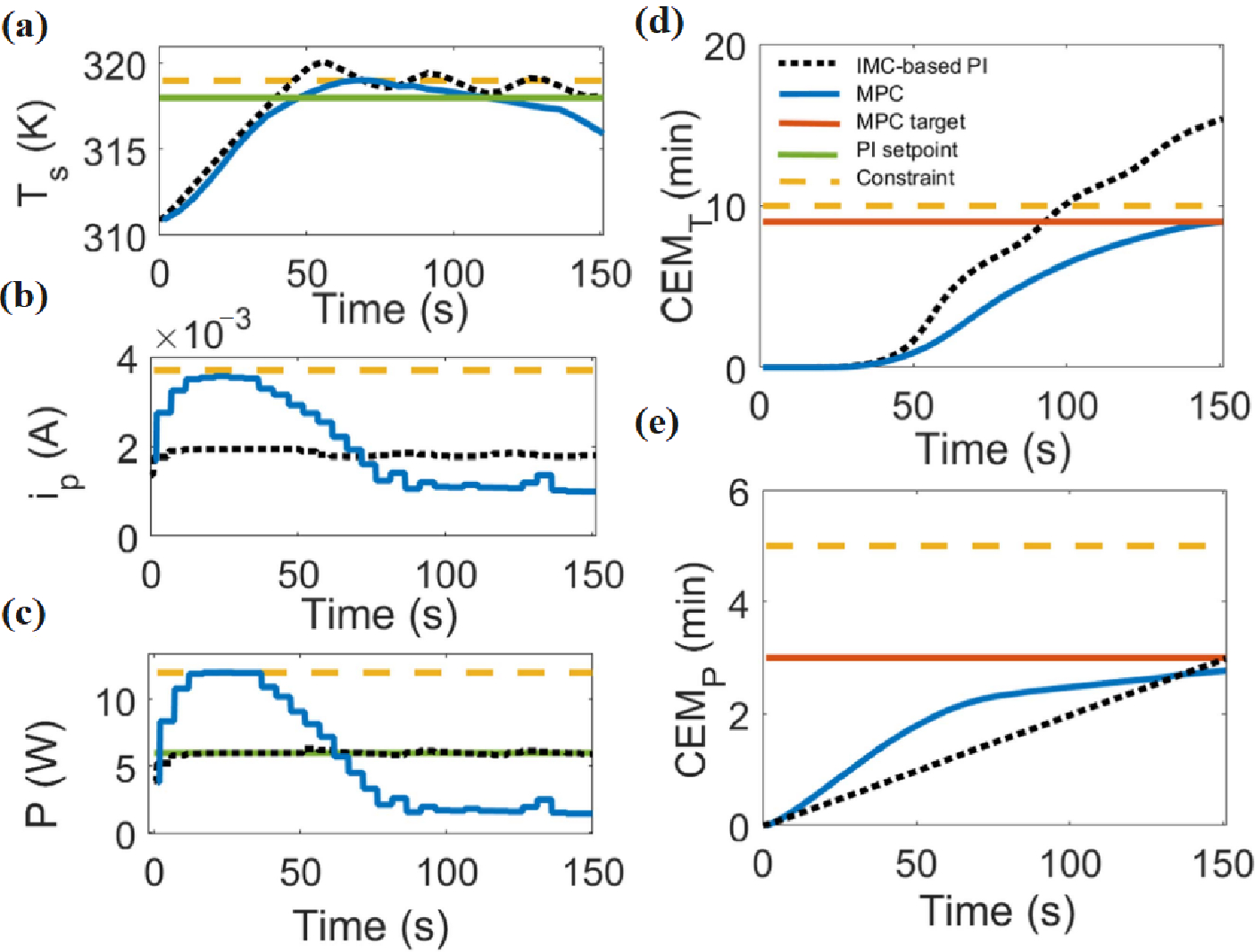
Closed loop simulation results for MPC controller vs PI control system in Case Study II: (**a**) substrate temperature, (**b**) plasma current, (**c**) plasma power, (**d**) thermal CEM, and (**e**) nonthermal CEM. The black dashed line in each image represent the IMC-based PI response, blue is the MPC response, orange is MPC target, green is the PI setpoint, and the yellow dashed line is the constraint. Reprinted/adapted from Ref. [102], with permission from, Copyright (2017), IOP Publishing Ltd.

The second test would go on to track the thermal and non-thermal doses applied to a substrate with the objective of delivering a defined thermal and nonthermal energy dose to the substrate by the end of the treatment time of 150 s. Similar constraints were defined with the addition of accumulative time metric restraints not exceeding the defined dose calculation by the end of 150 s (CEM_*T*_ ≤ 10 min, CEM_*P*_ ≤ 5 min). The results can be seen in Figure 16 [102]. The MPC controller did not overshoot threshold constraints for any of the output parameters including the dose metrics. While at first the MPC controller responded with close to threshold value outputs upon ignition of the plasma, they soon tapered off and achieved the dose metric requirements for thermal and nonthermal parameters. PI control on the other hand overshot surface temperature thresholds and oscillated around the constraint bound. This oscillation of the signal subsequently produced an overshoot of the constraints for both dose metrics. In summary, MPC control was better suited to handle the constraints needed for plasma medicine applications and also deduced that the reasoning for PI controllers overshooting defined constraints was likely due to the nonlinear nature of plasma and the dose delivery mechanisms [102].

Ultimately PID control systems are designed based on linear, single-input–single-output descriptions of system dynamics. Gidon et al. showed that for plasma medicine applications a PI controller cannot handle the complex nature of APPJ dynamics between various jet inputs and plasma effects [102]. Dose accumulation and real-time diagnostics of the plasma device were concluded to be too complex for PID controller. MPC controller have the capabilities to manage these input and outputs and ultimately provide a good baseline for future plasma medicine applications, where safety is paramount. It is with this understanding Gidon et al. recommended MPC derived plasma control as the true starting point controlling plasmas for medical applications.

#### 4.2.3. MPC Derived Plasma Control

After it was demonstrated that several PI, and PID derived plasma control methods helped in the overall effective control of the plasma, it soon became evident to the low-temperature plasma (LTP) community and especially the plasma medicine community, that more control for safe and effective treatment was needed. PI and PID control helped in demonstrating control of voltage and current for initial dissipated power in short intervals. Yet, PI and PID control could not adequately maintain surface temperature, or thermal and non-thermal cumulative equivalent minute dose constraints over time [102]. Thus the next step towards more advanced control of the plasma treatment process would be in real-time diagnostics with MPC controllers.

One of the first experimental implementations of real-time diagnostics utilizing an MPC and a linear parameter varying (LPV) framework was conducted by Gidon et al. [111], following up on their previous study described above. A kHz-excited APPJ in helium was used for the basis of the real-time modelling where a Arduino UNO micro-controller read in outputs for substrate temperature, plasma intensity, applied voltage, and discharge current. The inputs that were manipulated by the controller were flow rate and applied voltage, as in the previous study. Since the output variables exhibit nonlinear behavior, Gidon et al. explored whether the LPV-MPC control approach would yield accurate results when accounting for non-linear behavior of the output parameters. In their experiment, two cases were tested: (1) LPV-MPC setpoint tracking for surface temperature and plasma intensity with one and two scheduling variables with varying distance and movement (disturbances), and (2) thermal dose delivery for a stationary APPJ tracking the thermal CEM dose. The experimental results were comparable to the simulated results in their previous work [102] showcasing the superiority of MPC control for thermal dose delivery. The first case experiment demonstrated two scheduling variables produced measured values more in line with the constraint setpoints for surface temperature and plasma intensity than a single schedule variable. The second case study provided experimental proof that MPC plasma control enables to stay within defined setpoint constraints and achieve the desired CEM thermal dose during plasma treatment. Overall, Gidon et al. showcased that LPV-MPC with two scheduling variables yielded deviations from the temperature setpoint that were less than 1 °C and less than 0.5 °C in the case of disturbance rejection, while the desired thermal dose was delivered in 4 min [111].

**Figure 17.**
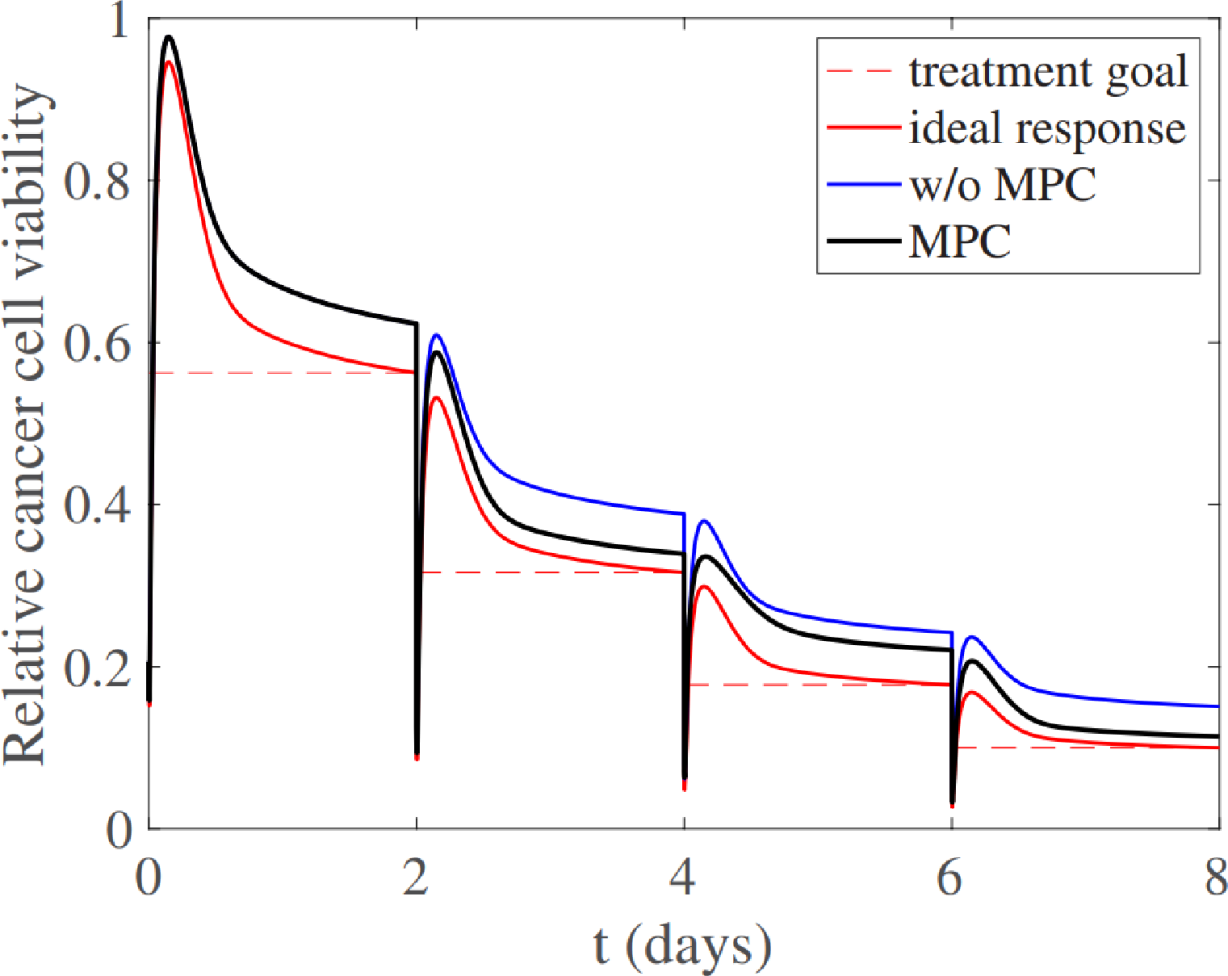
Model of the temporal response of relative cell viability. The dashed line shows the treatment goal. The red line illustrates an ideal case in which the accurate mathematical model for cell response is available. The blue curve shows the results when the accurate mathematical model is available but no MPC is used for optimal control. In black, MPC is introduced when the mathematical model is not ideal, showcasing the capabilities of MPC to mitigate modeling errors by optimizing the response. Reprinted/adapted from Ref. [112], with permission from, Copyright (2019), IOP Publishing Ltd.

Another example of MPC derived plasma control for medical applications was showcased mathematically by Lyu et al. where a model was developed to represent the dynamic response of cancer cells under CAP treatment [112], based on the data from in vitro experiments by Gjika et al. [100]. A mathematical model based on differential equations was developed to represent the experimental data where a kHz-excited APPJ was used for various treatment times. CAP-induced changes in metabolic activity, representing cell viability, was used as output. Plasma treatment duration and discharge voltage were chosen as inputs and an optimal control problem was formulated to minimize treatment time and voltage within the boundary of the desired cancer cell reduction. The authors included MPC to further generalize the optimal control problem to an optimal feedback framework, see Figure 17. The model developed by Lyu et al. establishes an MPC control strategy that can help in determining an effective treatment strategy for CAP treatments. Not only that, but this method of data accumulation and MPC control could be utilized in clinical practice for different cell lines to establish a method of complete reduction of cancer cell viability in patients [112].

MPC control has the capability, as demonstrated, to offer simulation based results on effective treatment strategies, as well as real-time diagnostic responses for in vivo plasma medicine treatments. MPCs outshine PI derived control methods since they can handle the multi-variable non-linear nature of plasmas, and should continue to be implemented in experimental and simulation based medical plasma experiments in an effort to reach a proposed treatment strategy.

#### 4.2.4. Neural Network/Machine Learning Derived Plasma Control

As a common theme in previously discussed studies, authors recommend that the next step towards progressing from established control methods lies in utilizing neural networks or machine learning to process the enormous amount of data to accurately predict the non-linear behavior of plasmas. Machine learning has had an enormous impact in many scientific disciplines and has also attracted significant interest in the field of LTP [113]. Trieschamnn et al. in their review of machine learning for plasma modeling and simulation detail how using database driven control, with the help of neural networks, can work in tandem with established control methods to facilitate real-time diagnostic capabilities for plasma processes. The strategy of utilizing neural network-produced databases for training and prediction has been realized for real-time diagnostics in few examples today, but they provide the most effective means of plasma control for medical applications yet seen. Up until neural networks demonstrated their capability for machine learning of non-linear dynamics, these aspects of plasma control were difficult to account for and predict by PID and MPC control in real-time. Examples of such behaviors include disturbances in the plasma transitioning from one surface to another, or this process occurring while one of the input parameters like gas flow or voltage were being manipulated. Non-linear derived MPC control can be produced offline through training models before implemented into real-time closed loop control based on measured diagnostic responses. It is with this in mind the conclusion was reached that embedded systems can account for the real-time and closed-loop feedback-based control, but the prediction of the nonlinear effects of the plasma and prediction of the substrate temperature can only be calculated based on deep neural-network (DNN) training methods [31].

**Figure 18.**
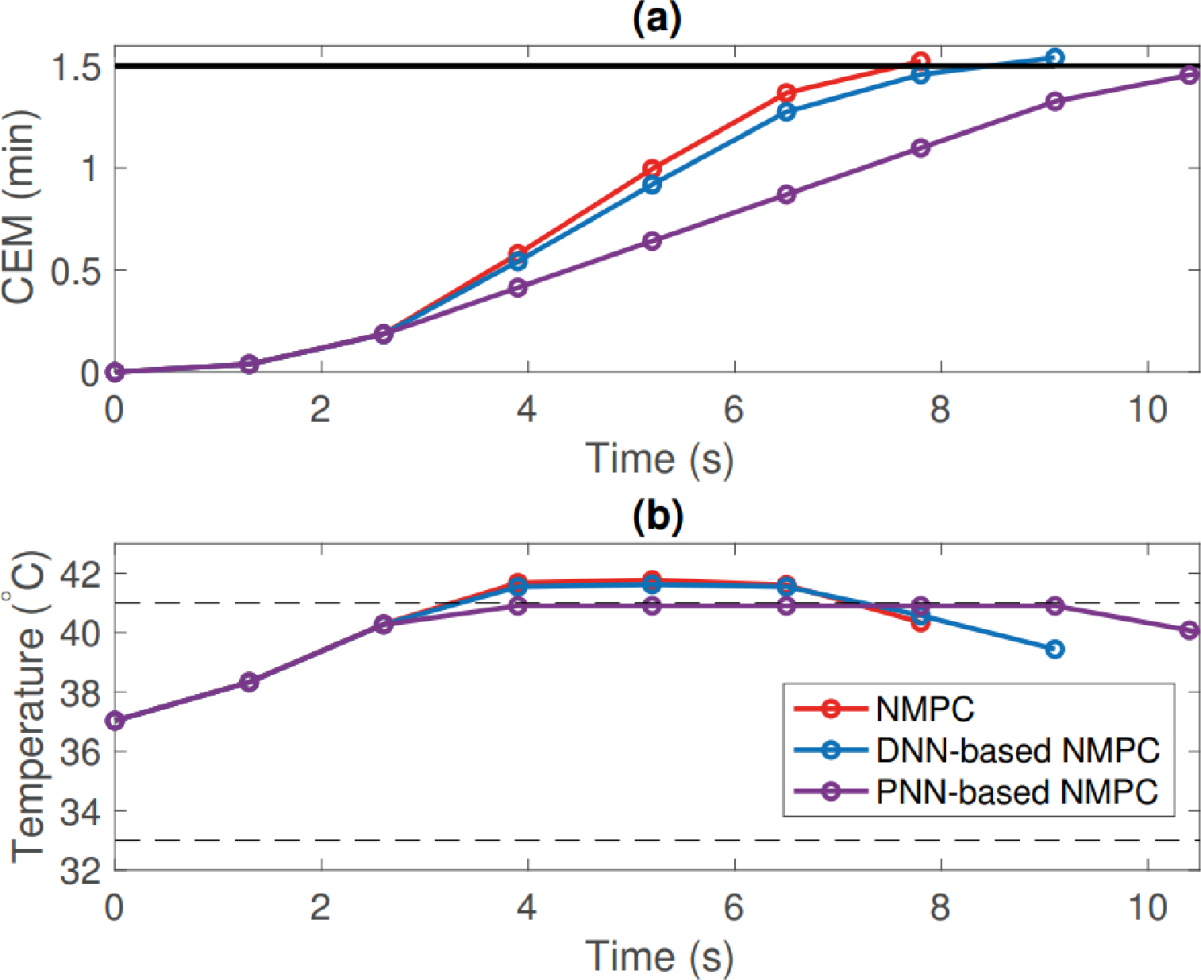
Closed loop simulation profiles of (**a**) CEM dose metric and (**b**) substrate temperature, comparing an explicit NMPC versus DNN and PNN-NMPC models. Reprinted/adapted from Ref. [31], with permission from, Copyright (2020), Elsevier.

Gidon et al. have presented early machine learning efforts for plasma medicine to determine rotational and vibrational temperature for substrate characteristics using OES linear regression in real-time [114]. From this information gathered in real-time, determination of the substrate could also be determined from the machine learning model. Yet due to the large quantity of data, off-line processing had to be done to determine the substrate feature of either glass or metal [114]. Deep-neural network trained models allow for this processing to be done in real-time, given the patterns in recognition can be identified during the process. One of the most recent and advanced uses of neural network control methods for plasma medicine applications was performed by Bonzanini et al. where an explicit NMPC was compared to DNN and project neural network (PNN) models within an NMPC control scheme for an APPJ system [31]. Within Bonzanini et al.’s experiment a kHz-excited APPJ with helium gas flow had controlled input of flow rate and applied power. The measured outputs for the system were substrate temperature, and total emission intensity through an Arduio UNO controller. To formulate the control problem the subspace identification took place by defining and accounting for all input and outputs along with weighting functions and disturbances with an uncertainly bound. The control definitions for this experiment utilized the thermal dose metric of CEM. An input constraint on the gas flow (0.8 slm ≤ q ≤ 10 slm) power (0.5 W ≤ P ≤ 5 W), and outputs of temperature (33 °C ≤ T ≤ 41 °C) and intensity (0 a.u. ≤ I ≤ 250 a.u.) were established. The DNN and PNN models were trained off 5000 experimental data samples and repeated five times. It was found that memory footprint of the DNN increased both with the number of nodes and layers. An optimal training model was set between 6–8 nodes for reduced mean squared error and optimal memory utilization [31]. The closed-loop simulation results, depicted in Figure 18 [31], showcased that DNN-NMPC provided a good approximation of NMPC law but temperature constraints were violated along with the explicit NMPC. PNN ensured constraints were not violated by modifying inputs more often and for a longer overall treatment time needed. PNN trades this for a worse control performance, by reaching the target CEM slower and prolonging treatment time by 35%, but maintained the constraints in all samples. In conclusion it can be highlighted that NPMCs have closed the gap in accounting for non-linear behaviors of plasma. The accumulative dose metric is a complex system when targeting substrates for applied plasma medicine research. Advanced control systems have the capability to solve this complicated problem, yet innovated methods like PNN may be the key to adapting plasma medicine devices to future clinical trials in the future.

#### 4.2.5. Reinforcement Learning Plasma Control

The real-time control of CAP devices is the ultimate goal for effective treatment in clinical settings, yet the best nonlinear predictive control schemes still have difficulty in modeling complex plasma surface interactions with multilayered substrates like biological samples. The main complexity lies in establishing quantified goals that the control scheme can quickly react to, all while balancing thermal, chemical, and electrical effects as outlined by Bonzaini et al. [37]. Therefore, targeting real-time monitoring of plasma parameters such as RONS, through multiple interactions with different substrates could, with enough trial-and-error, enable adjustment of treatment protocols to ensure effectiveness of the intended therapy [37]. This makes reinforcement learning an ideal candidate to be utilized for controlled medical plasma applications. For instance, in RL, no training data is provided via an algorithm. Instead, the RL agent is given a reward function and is left to learn the appropriate behavior through trial-and-error interactions with a dynamic environment [115]. The dynamic variables at play being the plasma, substrate, and transport of species would all play a direct part in successful development of treatment protocols.

RL has recently been demonstrated for plasma medical applications in regulating the thermal properties of APPJs on substrates with different thermal and electrical characteristics [36]. Witman et al. experimentally compared the ability of RL agents to track temperature and power setpoints through simulations and then compared this data experimentally on glass, aluminum, and polyimide substrates in a kHz excited APPJ. The APPJ’s applied voltage was regulated by a PI controller, and implemented on an Arduino UNO micro-controller through outputs of voltage and current. Temperature and power setpoints were tracked and employed to train three agents. The first agent was trained only on sampled data from the APPJ interacting with the glass substrate (G-RLC). The second agent was trained under the uncertainty cases where the glass was not detected (GU-RLC). Lastly, the third case was trained under examples from the APPJ interacting with each substrate (E-RLC). Witman et al. highlighted that the basis to which the RL was conducted was based upon four principles: (i) generating simulated data and/or collecting, (ii) evaluating the reward for the training data based on a user-specified control objective, (iii) providing reinforcement to the established model, and (iv) updating the RL agent to improve its performance based on the feedback [36]. Based off these principles, Witman et al.’s results showcased that each substrate, independently for the E-RLC agent, closely followed and matched the temperature and power setpoints. The G-RLC trained agent performed well when only treating glass, but for more conductive substrates like aluminum and polyimide it was not well maintained [36]. The E-RLC agent was 33% more efficient and resulted in 50% less input responses. Interestingly, the setpoints for the conductive substrate even with E-RLC, resulted in oscillatory temperature and power responses the more conductive the substrate was. Whitman et al. hypothesized this was due to the conductive substrates exhibiting thermal dynamics that were highly sensitive to changes in the input power.

Overall, it was determined that without dynamic randomization of various plasma-substrate trained models, RL controlled real-time experiments will not be as effective when it encounters a substrate that has significantly different dynamics than the agent it was trained on. The major drawback of this conclusion would be in the transition to patient treatments, as simulated models would need to be produced for training purposes before in-vivo experiments could be employed without inherent safety risks. Yet, RL was found to be very successful in terms of correctly analyzing and integrating more complex sensory information, retroactively, during real-time experiments than previously employed DNN models.

RL was also used by Hou et al. to find a safe exploration for adaptive plasma cancer treatment given the drawbacks of needing trained agents for targeted applications. By using in-vitro empirical data constructed through a Gaussian process, generalization of cancer responses to plasma treatment parameters were obtained. By using a Markov decision matrix, RL could help in determining an active value function (Q-learning) that could be trained under defined dynamic condition modeling. The Q-learning could then produce an optimized treatment policy that could reduce cell viability with a specific number of plasma treatments [104]. By teaching the RL agent the cancer dynamics and establishing an active value function, less aggressive treatments with fewer uncertainties could be achieved for cancer cell treatments [104].

#### 4.2.6. Personalized Plasma Control

With all of the possible control schemes and dynamic approaches to account for with the nonlinear behavior of plasmas, recent approaches employ actively tailoring the control method towards the patient or substrate retroactively. The newly founded approach labeled as “personalized control” has produced an adaptive control model that was explored earlier in this review (Section 3.1.1), but in the context of retroactively calibrating the plasma process to avoid drifts during the treatment. Yet, by configuring this approach, of Bayesian optimization, towards personalized and point-of-care plasma medicine the ability to actively adjust treatment parameters with complex substrates becomes attainable as demonstrated by Chan et al. Within Chan et al.’s work a DNN approximated MPC is utilized for controlling the power and flow rate of an APPJ to deliver a desired amount of plasma effects, as quickly as possible, without violating comfort and safety constraints [103]. Unlike BO which can be used to optimize the active process, a multi-output BO (MOBO) can be used to adapt and train DNN parameters for real-time applications by the MPC scheme. Each step of the closed-loop trajectory is a solution and represents a suitable situation in which the closed-loop system is likely to operate [103]. The results demonstrated MOBO could efficiently explore the parameter space of a DNN trained controller in closed-loop simulations to identify and adjust the treatment parameters to stay within acceptable treatment times in relation to the thermal dose being provided.

Preference-guided BO is another form of BO that was utilized by Shao et al. to offer statistically faster convergence, and computes solutions that better reflect user preferences versus RL where the true performance of a control policy becomes evident only after it has been applied [116]. By adopting preference-guided BO for plasma medical applications the ability to overcome the challenge of operating with limited data availability can be achieved. Preference-guided BO was even shown to outperform multi-output BO by targeting these optimal policies [116]. Within their study Shao et al. utilized an APPJ to treat heat and pressure sensitive bio-materials. The specific thermal dose metric of CEM was utilized to track the effective delivery of a thermal output to the substrates in the quickest possible time. A robust MPC strategy was employed to handle the uncertainties in the system, while the optimal state input variables were handled by a DNN. By minimizing treatment time, Shao et al. attempted to help overcome the drawbacks of RL in which limited data can be acquired to develop a well equipped agent, as well as the challenge of substrate-to-substrate performance variability. The solution to this problem, as defined by Shao et al., is by designating a quickly searchable user preference guideline that a MOBO can then utilize to override conflicting objectives. The results of providing user preferred guidelines allowed co-active feedback for higher utility in their experiments. Ultimately this method showcased that it could quickly reduce the required treatment time while still satisfying the temperature constraints in comparison to traditional MOBO and BO. Overall, the preference-guided BO could achieve tailored responses, requested by the user, in an effective way during treatment.

## 5. Discussion

### 5.1. General Impact

**Figure 19.**
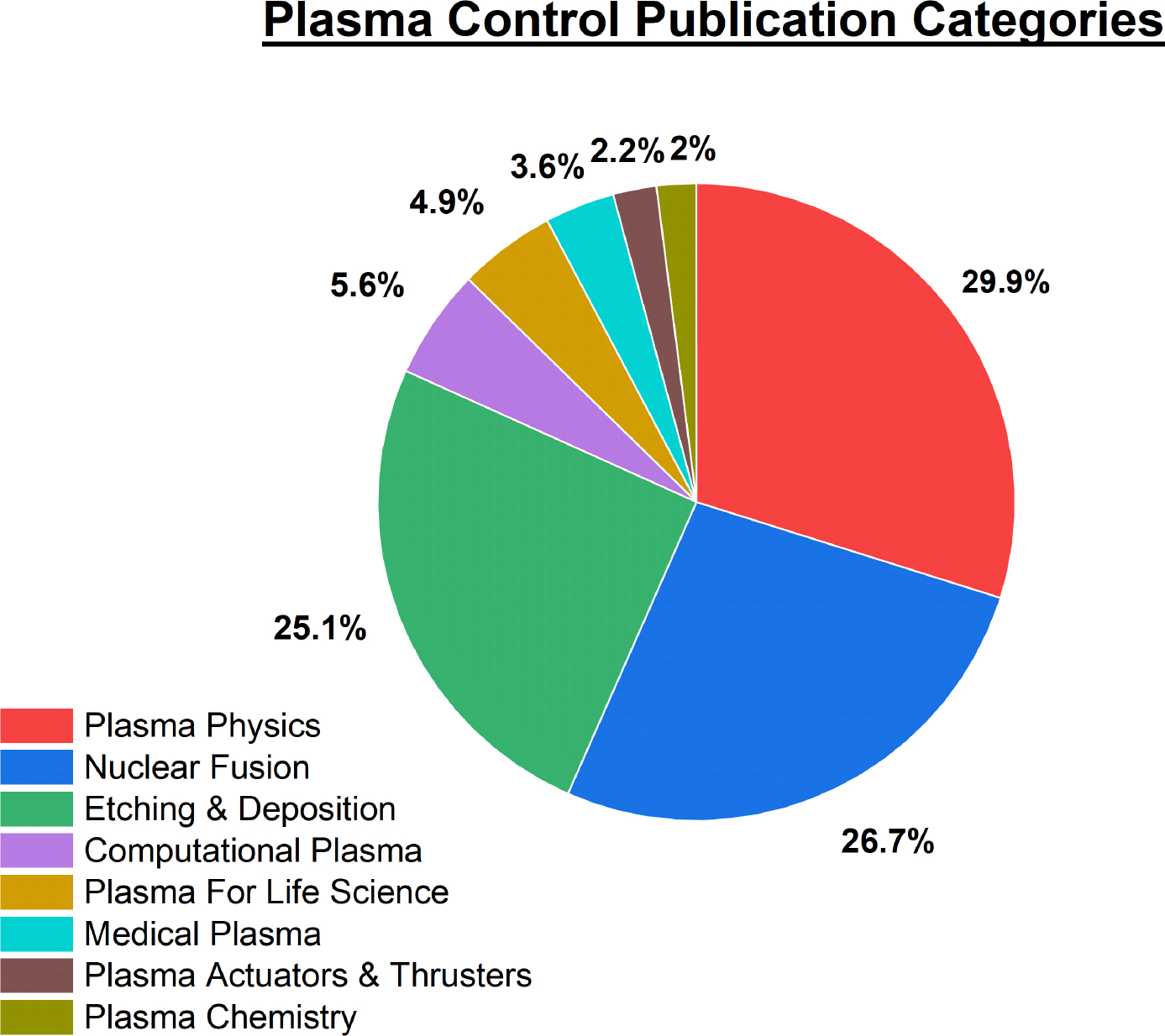
Plasma control publications published since 1940 based on polled queries filtered through the web of science database. Plasma publications are categorized based off their subsequent topic or field of application.

It can be deduced, the fundamental control of plasmas across different fields has lead to several breakthroughs in their applications in the modern era that are evident in daily life. With significant breakthroughs in semiconductor manufacturing, due to plasma control, the prospects of this technology being brought to the forefront of the medical field are quite tenable. While the early onset of plasma applications focused on controlled fusion research, these early experiments on the fundamental nature of plasmas led to large-scale efforts into cold plasma processes that could be utilized for broader applications. Figure 19 categorizes the plasma control publications that have been released since the 1940s. A large majority of the papers published focus on the fundamental understanding of the nature of plasmas from a physics perspective, with the next two most published categories being fusion, and etching and deposition research. The ensuing categories highlight the new and growing fields in plasma control research. Computational plasma research has lead to contributions to each of the other categories listed by making advancements in understanding the processes occurring within the plasma, the plasma-material interactions, and the behavior of plasma itself under different conditions [117]. Following computational plasmas, the next largest area of focus in controlled plasmas is in life sciences applications. Life science applications have benefited significantly from the advancements in plasma control that can be seen in examples of tailored plasma treatments of wastewater [118], agriculture [119], and air purification [120]. Many of the experiments regarding life science applications have not incorporated real-time diagnostics and advanced controller schemes yet, but with the onset of recent successes in machine learning this area of plasma control is likely to increase in the coming years. Medical plasmas was the next category and is still untapped in many regards as demonstrated in previous sections. Plasmas for aerospace and renewable energy via plasma actuators and thrusters have gained renewed interest in recent years thanks to DBDs being utilized to produce propulsion or reducing aerodynamic load fluctuations [121]. Thanks to closed-loop control strategies these plasma applications have become a reality due to the the enhanced capabilities in power management in igniting plasmas, as well as the ability to retroactively respond to plasma behavior in aerodynamic conditions. The least published on, but arguably most vital category towards implementation of plasma medicine applications, is plasma chemistry. Plasma chemistry control research has thus far focused on the quantification and response of RONS to plasma-defined parameters. More data is needed in order to determine device-specific differences and to draw conclusions regarding different chemistry regimes for various applications, e.g., the treatment of different diseases in plasma medicine. Collaboration in combining these two research areas with the development of recent advances is inevitable, but severely needed when progressing towards future research.

### 5.2. Current Challenges

Currently, within the realm of plasma medicine, only a few methods have been implemented into real-time controller mechanisms. Temperature measurements, deposited power, and OES during operation have been some of the first cases of successful implementations of measured outputs for controllers. Machine learning methods that haven’t been implemented into real-time diagnostics, but have produced outcome parameters, are Fourier transform infrared spectroscopy (FTIR) measurements [122], fluid modelling, and substrate target feedback [123]. Each of these diagnostic methods have yet to be incorporated and tested in experimental applications. Validation of different controller methods will be vital moving forward if plasma medical therapy is to be achieved. Across fusion and semiconductor manufacturing, innovative approaches have been taken into acquiring diagnostics that have yet to be realized within plasma medical control approaches. Active monitoring of plasma impedance during treatments is one such method that could actively benefit the control process moving forward.

Real time feedback control is also still lacking in specific areas in terms of accurately accounting for generated species. Thus an area of plasma control that needs to be investigated further for the success of medical based applications is within plasma chemistry. Considerable effort within recent years has taken place in the computational space of low temperature plasmas to help determine the density and type of RONS that are produced in differing plasma geometries and scenarios [5]. Lietz et al. highlighted how global modelling of RONS production in plasma liquid interactions was possible for known plasma reaction mechanisms on large timescales. It was noted that the investigated method may not be suitable for all geometries. Future efforts should be placed in computationally simulated plasma outputs to help in the determination of a plasma dose for implementation into real-time micro-controller diagnostics. By adjusting input parameters of the plasma, simulated RONS productions could hold the key to tailoring control mechanisms to selectively produce the desired amount of RONS for plasma medical applications. Waveform tailoring is one such example in which controlling the electron-driven plasma chemistry can produce differing metastable densities. This method is already in practice in the etching industry today with the potential to be implemented into medical applications. Recently Korolov et al. demonstrated experimentally and computationally that voltage waveform tailoring enhanced the generation of helium and atomic nitrogen metastables in a radio frequency *μ*-APPJ configuration [124]. It was discovered that by manipulating the amount of consecutive driving harmonics and gas flow, a break in the symmetry of the spatio-temporal electron power absorption dynamics of the plasma occurred [124]. This break in symmetry caused a reduction in the DC-self bias of the system, increasing the energy tail of the electron energy probability function (EEPF). By being able to manipulate the EEPF, metastable generation can be enhanced to a desired amount to allow access to plasma chemistry regimes that were previously inaccessible [124].

Additionally, DBD electrode configurations are one of the few plasma systems that have not been directly implemented into one of the many controller methods mentioned. Self-tuning power supply applications have been applied, but none for medical applications have surfaced. APPJs have been the dominate force in real-time, closed-loop plasma diagnostic experiments due to the ease of diagnostic capabilities and similarities of devices across the field. Real-time diagnostics for DBDs have been a challenge for the field due to the variation in electrode configuration, dielectric material utilized, and accounting for how to accurately perform diagnostics on each of these configurations. For example, since a volume DBD interacts directly with the target being treated, the distance of treatment alone can effect the capacitance of the circuit impacting applied plasma parameters. This interaction with DBD control extends to the difference in plasma surface interactions ranging from liquids, solids, and the varying conductivity and permittivity of each surface. It is with each of these considerations that DBDs, especially volume DBDs, have yet to have a direct real-time control method implemented in an automated control scheme configuration.

MPC control methods provide a solid staring point for controlling plasmas for medical applications. Yet a deep interdisciplinary knowledge on control systems, the non-linear dynamics of plasmas, and the cellular and chemical responses of the targets being treated need to be understood. Collaboration across multiple fields of study will be necessary in order to achieve the aims of plasma medicine in clinical practice with specific dose requirements for given afflictions. Collaboration and consolidation into efforts like machine learning and AI can help bridge the gap for each of these specializations to handle the correlations in data presented in studies like Gjika et al.’s report [100].

### 5.3. Recommendations

**Figure 20.**
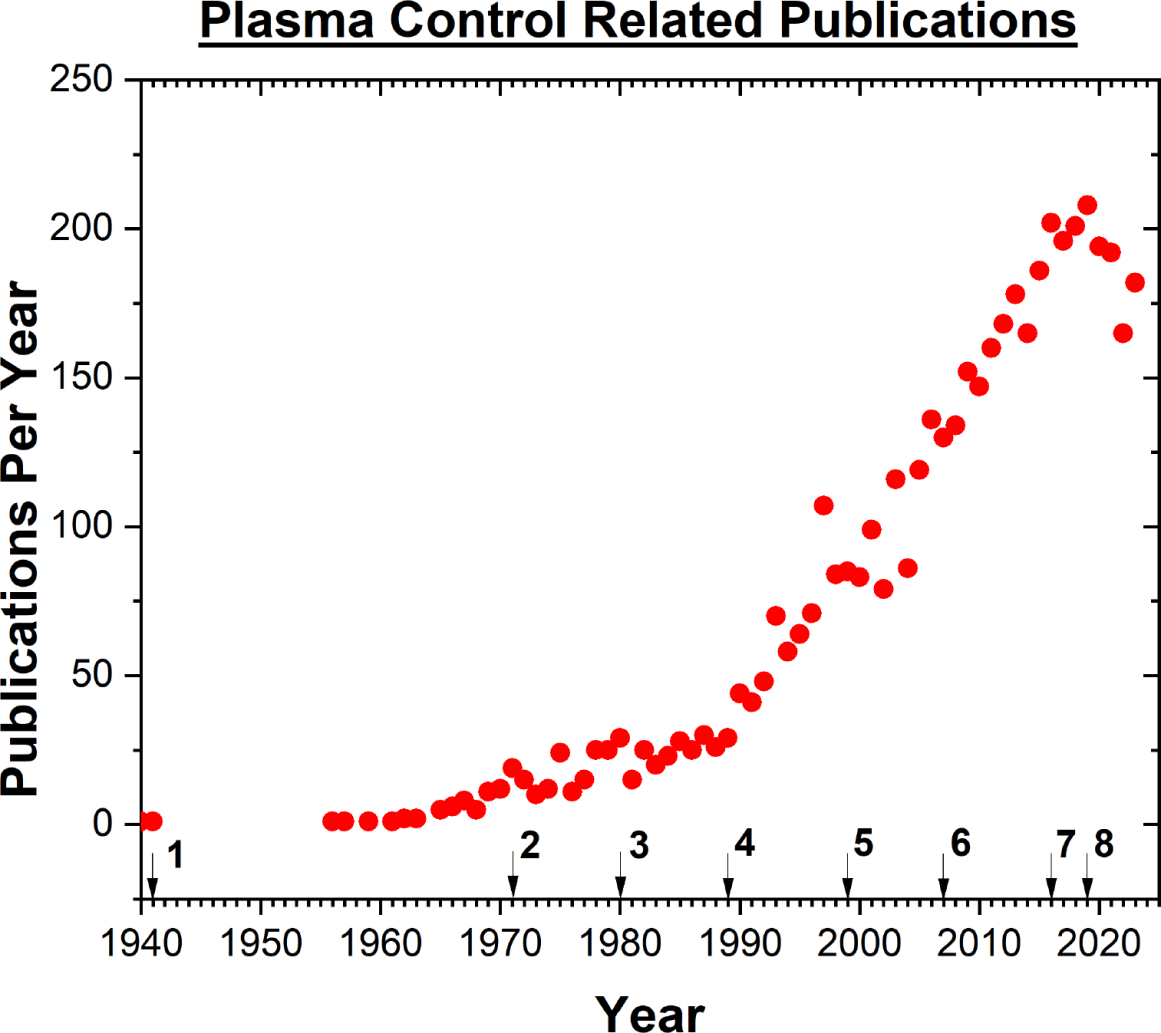
Publications of peer reviewed papers per year from 1940 to 2023 listed in the web of science database with the key phrase ‘Plasma control’. Red bullets indicate the amount of ‘Plasma control’ publications for a given year. Arrows indicate dates of significant plasma control papers that advanced the field: (1) Leimberger [125], (2) Brown et al. [126], (3) Hirobe et al. [127], (4) Tsai et al. [128], (5) Stevenson et al. [45], (6) Moreau et al. [129], (7) Bruggeman et al. [130], (8) Gidon et al. [114]

Currently a majority of the plasma control investigations have been, and still are, dominated by fusion, etching, and the fundamental nature of plasma research. In recent years, control of plasmas for other applications such as the life sciences or medical applications have become more prevalent. The growth of literature in the field of plasma control since its conception in the 1940s is depicted in Figure 20. With improved understanding of plasmas and the methods to control them, next steps should involve more consideration into correlating plasma outputs with cellular response to enable informed decisions on the selection of outputs, inputs, and the best control scheme. It was shown that non-linear derived forms of control are necessary, and weighting functions for different species or constraints might have to be identified before plasma control in medical treatment becomes a reality.

Several of the challenges mentioned above originate in the use of different devices, input parameters, and outputs for the given plasma applications. With numerous inputs and devices to consider, the most logical path towards the consolidation and reproducibility of a universally defined plasma treatment is through individual algorithmic based feedback controllers. A colloquial database that can contribute to an overall neural network trained model under the identification of specific parameters given a targeted application such as plasma medicine could be developed moving forward. Standardization of devices for surface and volume DBDs should also be considered as was done in the case of the COST jet. The COST Jet was created to set a comparison standard between different groups in Europe and the world [131]. Standardization would help in resolving the copious amounts of data that is needed in determining optimal parameters for applications from wound healing to cancer treatment for DBDs and can only help in improving the quality and reproducibility of data moving forward.

In conclusion there are many areas of plasma control in medicine that are yet to be explored. Looking at where the industry has come from, it can help guide experimental methods moving forward. Working towards advancing implementation of acquired data into machine learning algorithms will provide prediction models to help in advanced medical applications once a database has been established.

## 6. Conclusions

Within this review an exploration into the history and conception of control engineering was explored. An introduction into the basics of control engineering was provided in an effort to layout a solid understanding of the control mechanisms needed, and in place today, to control a multi-variable system like plasma. Established methods of controlling plasmas were highlighted such as voltage, and current, while less known methods like substrate and external signal control were provided for additional context. A brief history on plasma medicine was then followed by examples of plasma control within the field, detailing different control schemes that have achieved success. Other areas of plasma control like etching and deposition were highlighted as several of the established control methods used today originate in these sub-fields. Lastly, a discussion was provided to highlight the current challenges and recommendations for experiments and methods of control moving forward. From this reviewed publication it has been shown that plasma outputs are typically non-linear in nature and require advanced control schemes to be modelled and predicted correctly. Only dedicated control schemes that can handled multi-variable inputs and outputs can provide an avenue towards safe and effective treatment of patients in the future. Neural networks, and machine learning capabilities have advanced the control field significantly by providing a method for processing large amounts of measured data. Machine learning in some cases has helped in creating more efficient control schemes than the established ones. The future is bright for plasma medicine control due to these advancements and continued effort in these new fields will only accelerate the implementation of real-time diagnostics in controllers for on-going experiments moving forward.

## Data Availability

Not applicable as this manuscript does not report new data.
